# Controlling asymmetric transmission phase in planar chiral metasurfaces

**DOI:** 10.1515/nanoph-2021-0558

**Published:** 2021-12-22

**Authors:** Ranran Zhang, Qiuling Zhao, Xia Wang, Kai Ming Lau, Tsz Kit Yung, Jensen Li, Wing Yim Tam

**Affiliations:** Physics Department, Optoelectronic Materials and Technologies Engineering Laboratory, Shandong, QingDao University of Science and Technology, Qingdao, Shandong, China; Department of Physics and William Mong Institute of Nano Science and Technology, Hong Kong University of Science and Technology, Clear Water Bay, Kowloon, Hong Kong, China

**Keywords:** asymmetric transmission, birefringent material, controllable phase, planar chiral metasurface

## Abstract

Metasurfaces with ultrathin artificial structures have attracted much attention because of their unprecedented capability in light manipulations. The recent development of metasurfaces with controllable responses opens up new opportunities in various applications. Moreover, metasurfaces composed of twisted chiral structures can generate asymmetric responses for opposite incidence, leading to more degrees of freedom in wave detections and controls. However, most past studies had focused on the amplitude responses, not to mention using bi-directional phase responses, in the characterization and light manipulation of chiral metasurfaces. Here, we report a birefringent interference approach to achieve a controllable asymmetric bi-directional transmission phase from planar chiral metasurface by tuning the orientation of the metasurface with respect to the optical axis of an add-on birefringent substrate. To demonstrate our approach, we fabricate planar Au sawtooth nanoarray metasurface and measure the asymmetric transmission phase of the metasurface placed on a birefringent sapphire crystal slab. The Au sawtooth metasurface-sapphire system exhibits large oscillatory behavior for the asymmetric transmission phase with the tuning parameter. We confirm our experimental results by Jones matrix calculations using data obtained from full-wave simulations for the metasurface. Our approach in the characterization and light manipulation of metasurfaces with controllable responses is simple and nondestructive, enabling new functionalities and potential applications in optical communication, imaging, and remote sensing.

## 1 Introduction

Conventional optical devices made of natural materials have weak optical properties and thus they are usually bulky to achieve noticeable effects. To overcome this drawback, metamaterials made of artificial structures with sizes much smaller than the wavelengths of interest have been developed to provide a compact solution for the manipulation and control of light [1], [2], [3]. Recently, metasurfaces [4], [5], [6], [7] (two-dimensional versions of metamaterials) have drawn much interest because of their flexibility in amplitude and phase controls, and also the simplicity in fabrications and large-scale productions [8], [9], [10]. Due to the advances in nanotechnologies, metasurfaces can now be designed to achieve specific optical properties for device applications such as beam deflectors [11, 12], ultrathin metalenses [13, 14], vortex beam generators [15, 16], asymmetric metaholograms [17, 18], etc. Despite a large number of work on metasurfaces that have been reported [19, 20], a grand challenge metasurfaces/metamaterials face is their static functionalities; that is the materials, structures, sizes, etc. are fixed and cannot be changed after the fabrication, thus considerably limiting the degree of freedom for full-wave controls and applications. Substantial efforts [21] are now dedicated to overcoming the static operation of metasurfaces to achieve active control by e.g. mechanical [22], electromagnetic [23], and even thermal [24] tunings.

As most metamaterials are not sensitive to the handedness of the incident light (labeled as achiral), chiral metamaterials are sensitive to the handedness of the incident light and thus can add more degrees of freedom for wave controls and manipulations. Chiral metamaterials have surpassed ordinary natural chiral materials in applications as they have much stronger chiral effects [25, 26]. These chiral metamaterials, especially the two-dimensional versions labeled as chiral metasurfaces, have great potentials in wave controls and manipulations as they, in addition to the handedness, are also sensitive to the direction of the incident light, known as asymmetric transmission [27, 28]. Chiral metasurfaces have attracted much interest recently because of the potential for practical applications and devices like optical isolators [29], circular polarizers [30], and anisotropic chiral imaging [31] have been experimentally demonstrated. Moreover, metasurfaces/metamaterials with tunable chirality such as controllable optical activity (OA) [32], circular dichroism (CD) [33], circular conversion dichroism (CCD) [34], and asymmetric transmission (AT) [35] have also been reported. Despite these efforts, it is still challenging to fabricate tunable chiral metasurfaces/metamaterials for the optical range due to technical limitations. Moreover, the reported tunable chirality is based mainly on controlling the amplitude responses, and not much on controlling the chiral phase responses which is just as important in the control and characterization of chiral metamaterials, e.g. like the circular phase dichroism (CPD) reported recently for the characterization of chiral metasurfaces [36].

In this letter, instead of varying the elements of the metasurfaces, we take the approach of tuning the responses by an add-on external birefringent material to control the asymmetric transmission phase (ATP) of planar chiral metasurface using birefringent interference (BI) induced by the difference in the refractive indexes for the electric field components along with the ordinary and the extraordinary axes of an add-on birefringent substrate. The bi-directional transmission phase, and hence the ATP, of the planar chiral metasurface, can be extracted via the BI oscillations which can be tuned by varying the angle between the orientation of the metasurface w.r.t. optical axis of the birefringent substrate. Importantly, the transmission phase is not sensitive to the amplitude of the signal [36] as compared with the traditional AT characterization for chiral materials. Moreover, as we are interested in relative phase measurements, e.g. w.r.t. the phase of the birefringent substrate, systematic backgrounds or errors can be canceled to achieve more robust results [37].

To demonstrate our approach, we fabricate planar chiral metasurface consisting of Au sawtooth nanoarrays on a nonbirefringent substrate using e-beam vapor depositing and focus ion-beam techniques. A uniaxial birefringence a-cut sapphire crystal slab with the optical axis on the plane of the substrate surface is added to the metasurface to form a metasurface-sapphire system for transmission phase measurements using the BI approach as reported recently [37]. The transmission phase of the system is tunable by varying the angle between the orientation of sawtooth nanoarray w.r.t. the optical axis of the sapphire slab. (More details can be found in Section 3 of the Supplementary Material.) Using this BI approach, we obtain a controllable ATP for our Au sawtooth metasurface-sapphire system with more robust responses compared to that of using the traditional AT measurements. To provide support for our experimental results, we use a Jones matrix method to calculate the responses of the metasurface-sapphire system using data obtained by performing full-wave simulations for the Au sawtooth metasurface and obtain good agreements. Moreover, our approach is nondestructive and can be applied remotely to samples fabricated on ordinary substrates, opening up new opportunities in optical communication, imaging, and remote sensing applications.

## 2 Controllable asymmetric transmission phase

**Figure 1:**
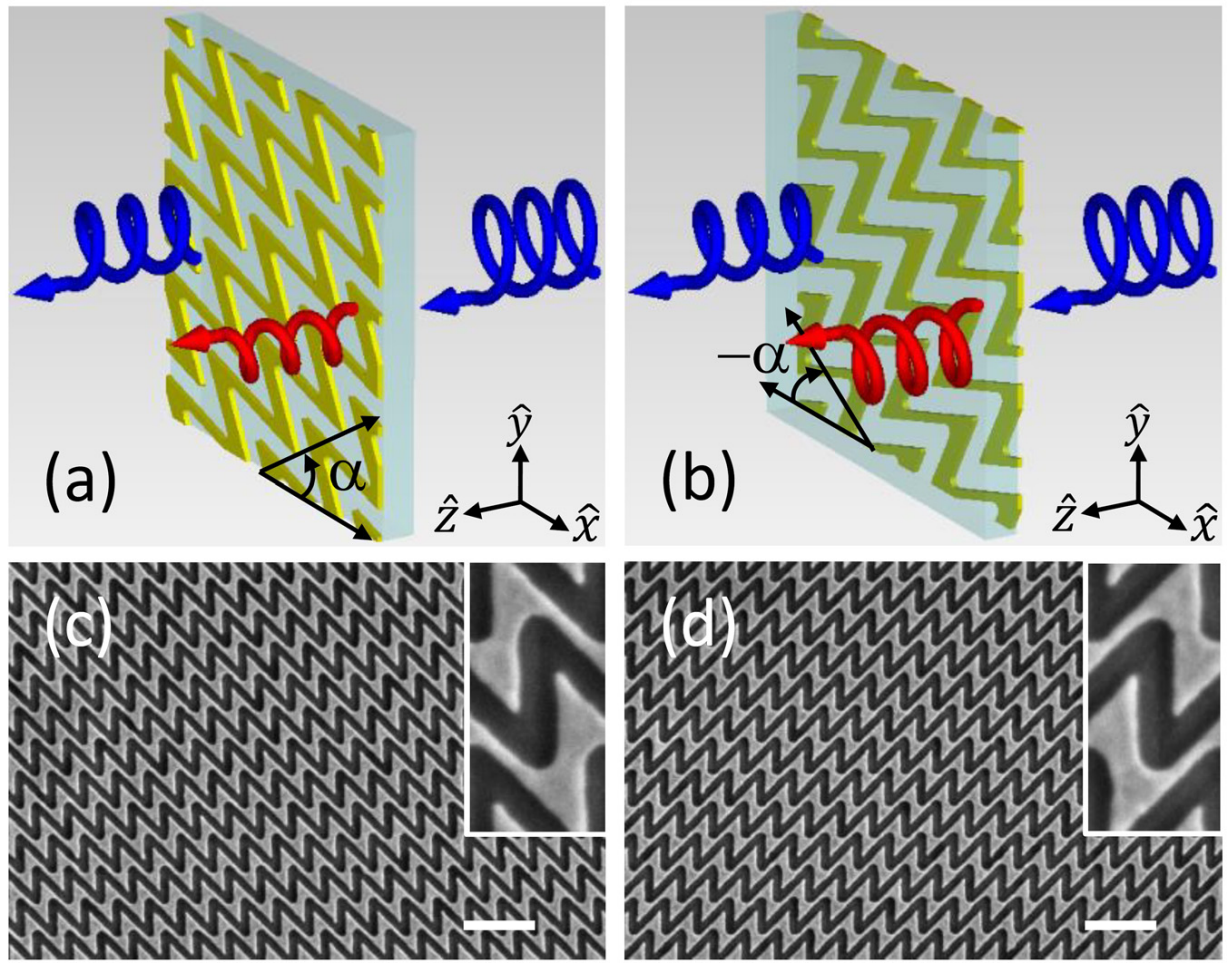
Schematic of bidirectional transmission of planar sawtooth chiral metasurfaces. (a) and (b) Transmission of circularly polarized light (RCP, +) for (a) forward and (b) backward (flipping sample along the $\hat{y}$ -axis.) direction of a chiral system consisting of a planar Au sawtooth metasurface (yellow) and an *L* = 2 mm thick a-cut birefringent sapphire slab (cyan) with an optical axis fixed along the $\hat{x}$ -axis. The blue helical arrows are for RCP and the red helical arrows are for LCP. The base angle ( α) between the Au sawtooth nanoarray of the metasurface and the optical axis of the sapphire plate can be controlled by simply rotating the metasurface w.r.t. the sapphire slab. (c) and (d) SEM images of single-layered **N**- and И -type Au sawtooth metasurfaces, respectively. The white scale bars are 1 μm. The width of the Au sawtooth is about 115 nm for the vertical bar and about 100 nm for the slanted bar for the **N**- and И -type metasurfaces. Insets in (c) and (d) are enlarged images of the unit cells of the chiral metasurfaces.

We choose single-layered Au sawtooth nanoarray gratings consisting of **N** and **И** basic units/“atoms” as reported earlier [38] to demonstrate the asymmetric transmission. The sawtooth gratings are similar to the nano-arrays from other groups [39], [40], [41], [42]. Note that the Au sawtooth nanoarray metasurface is periodic and consists of “meta-atoms” with the same orientation such that the transmission response will have a uniform wavefront as we focus only on the asymmetric transmission responses. The Au sawtooth metasurface can be modified to produce a spatially dependent wavefront by combining “meta-atoms” (here **N** or **И**) with different orientations to form “super-cells” or patterns as reported recently, using similar “meta-atoms”, for achieving gradient geometric phase [40] and the generation of orbital angular momentum [41]. In principle, combining “meta-atoms” with different orientations, sizes, and shapes, into a specific format (periodic or aperiodic) can achieve spatially dependent wavefronts [19, 20]. Figure 1a shows a schematic diagram (ignoring the metasurface substrate) for a forward ( $+\hat{z}$ ) circular polarization incidence, here right-handed circularly polarization (RCP, + ), on an **N**-type Au sawtooth chiral metasurface (yellow color)–birefringent sapphire (cyan color) system. (We define our convention of the chiral metasurface as viewed toward the incoming light.) The incoming light first enters the sapphire slab from the air–sapphire interface, passes through the Au sawtooth metasurface, and then exits from the metasurface–air interface. The transmitted light will possess the incident polarization (RCP) component as well as the left-handed circularly polarization (LCP, − ) component due to polarization conversion by the Au sawtooth metasurface [42, 43]. For the backward incidence, instead of light incident from the − $\hat{z}$ -direction, the metasurface system is simply flipped along the $\hat{y}$ -axis but keeping the incident direction unchanged as indicated in Figure 1b. Now the incoming light will first enter from the air–metasurface interface, passes through the sapphire plate, and then leaves the system from the sapphire-air interface. Note that now the metasurface will be a mirror image of the original pattern, namely an **И**-type sawtooth nanoarray grating when viewed toward the incident light. Importantly, the transmission amplitudes and also the phases, for different polarizations, are in general different for the forward and backward incidence, as represented by the sizes of the helical arrows for the transmitted RCP (blue color) and LCP (red color) light in Figure 1a and b due to the difference in the circular polarization conversion efficiency of the metasurface. Moreover, the transmission for the metasurface-sapphire system also depends on the base angle (*α*) between the optical axis (defined as the $\hat{x}$ -axis) of the birefringent sapphire slab and the lattice orientation of the Au sawtooth nanoarray grating such that it can be used as a tunable parameter for the sawtooth metasurface-sapphire system.

We use the Jones matrix approach to model the propagation of waves for our Au sawtooth metasurface-sapphire system [28]. (More details about the Jones matrix for the metasurface-sapphire system can be found in Section 1 of the Supplementary Material.) For waves propagating in the positive $\hat{z}$ -axis the electric field E can be written as:

(1) $$E=\left(\begin{array}{l} E_{x}\left(t\right)\\ E_{y}\left(t\right) \end{array}\right)=\left(\begin{array}{l} E_{0x}e^{i\phi_{x}}\\ E_{0y}e^{i\phi_{y}} \end{array}\right)e^{i\left(kz-\omega t\right)}\text{,}$$

where $E_{0x/0y}$ is the amplitude and *ϕ*_*x/y*_ the initial phase delay, *k* = 2π/*λ* is the wave vector with *λ* the wavelength and *ω* is the angular frequency. The transfer matrix in the Jones representation for a metasurface at normal incidence can be written in circular polarization bases as [39]:

(2a) $$t^{l,F}\left(\alpha \right)=\left(\begin{array}{ll} t_{++}^{l,F} & e^{-i2\alpha }t_{+-}^{l,F}\\ e^{i2\alpha }t_{-+}^{l,F} & t_{--}^{l,F} \end{array}\right)$$

for the forward (*F*) incidence and

(2b) $$t^{l,B}\left(-\alpha \right)=\left(\begin{array}{ll} t_{++}^{l,B} & e^{i2\alpha }t_{+-}^{l,B}\\ e^{-i2\alpha }t_{-+}^{l,B} & t_{--}^{l,B} \end{array}\right)$$

for the backward (B) incidence, where *l* = N/N^−1^ denotes our planar **N**-/**И**-type Au sawtooth nanoarray chiral metasurface. α/−α is the forward/backward base angle (positive for anticlockwise and negative for clockwise) between the lattice/“atom” baseline of the sawtooth nanoarray and the $\hat{x}$ -axis. $t_{ij}^{l,F/B}=\left|t_{ij}^{l,F/B}\right|e^{iArg\left(t_{ij}^{l,F/B}\right)}$ is the transmission coefficient. The subscript indexes, *i* and *j*, of $t_{ij}^{l,F/B}$ are the outgoing and incoming light, respectively.

To obtain the transmission phase, we add a birefringent plate/slab (a-cut sapphire) to the metasurface as reported earlier for circular phase-dichroism measurements [37]. As sapphire is a uniaxial birefringent crystal, the refractive index for the ***E*** field component along the extraordinary (optical) axis ( $n_{\text{e}}$ ) is different from that of the ordinary axis ( $n_{\text{o}}$ ). Especially for an a-cut sapphire slab, the optical axis (chosen as the reference $\hat{x}$ -axis) is on the plane of the slab, and thus the Jones matrix for the a-cut sapphire slab at normal incidence can be written in circular polarization bases as:

(3) $$t^{\text{Sa}}=\left(\begin{array}{ll} \cos\left(\frac{\delta }{2}\right) & i\text{sin}\left(\frac{\delta }{2}\right)\\ i\text{sin}\left(\frac{\delta }{2}\right) & \cos\left(\frac{\delta }{2}\right) \end{array}\right)\text{,}$$

where δ = $\frac{2\pi }{\lambda }\left(n_{\text{o}}-n_{\text{e}}\right)L$ for *L* the thickness of the sapphire slab. Note that the transmittance of the sapphire slab will exhibit BI oscillations proportional to *L* and transmission phases $\phi_{ii}^{\text{Sa}}=0$ and $\phi_{ij}^{\text{Sa}}=\pm \pi$ for the co- and cross-polarizations (see Eq (S4) of the Supplementary Material), respectively, independent of the incident direction. Then, the total transfer matrix for the Au sawtooth metasurface-sapphire system for the forward ( $E^{l,\text{F}}$ ) and backward ( $E^{l,\text{B}}$ ) incidence can be written in circular polarization base as:

(4a) $$E^{l,\text{F}}\left(\alpha \right)=\left(\begin{array}{ll} E_{++}^{l,\text{F}} & E_{+-}^{l,\text{F}}\\ E_{-+}^{l,\text{F}} & E_{--}^{l,\text{F}} \end{array}\right)=t^{l,\text{F}}\left(\alpha \right)t^{\text{Sa}}=\left(\begin{array}{ll} t_{++}^{l,\text{F}} & {\text{e}}^{-i2\alpha }t_{+-}^{l,\text{F}}\\ {\text{e}}^{i2\alpha }t_{-+}^{l,\text{F}} & t_{--}^{l,\text{F}} \end{array}\right)\left(\begin{array}{ll} \cos\left(\frac{\delta }{2}\right) & i\text{sin}\left(\frac{\delta }{2}\right)\\ i\text{sin}\left(\frac{\delta }{2}\right) & \cos\left(\frac{\delta }{2}\right) \end{array}\right)\text{,}$$

(4b) $$E^{l,\text{B}}\left(-\alpha \right)=\left(\begin{array}{ll} E_{++}^{l,\text{B}} & E_{+-}^{l,\text{B}}\\ E_{-+}^{l,\text{B}} & E_{--}^{l,\text{B}} \end{array}\right)=t^{\text{Sa}}t^{l,\text{B}}\left(-\alpha \right)=\left(\begin{array}{ll} \cos\left(\frac{\delta }{2}\right) & i\text{sin}\left(\frac{\delta }{2}\right)\\ i\text{sin}\left(\frac{\delta }{2}\right) & \cos\left(\frac{\delta }{2}\right) \end{array}\right)\left(\begin{array}{ll} t_{++}^{l,\text{B}} & {\text{e}}^{i2\alpha }t_{+-}^{l,\text{B}}\\ {\text{e}}^{-i2\alpha }t_{-+}^{l,\text{B}} & t_{--}^{l,\text{B}} \end{array}\right)\text{.}$$

Furthermore, the transmittance of the metasurface-sapphire system can then be expressed in oscillating cosine functions as:

(5a) $$T_{++}^{\text{l,F}/\text{B}}=\frac{1}{2}\left\{A_{1/1}^{l,\text{F}/\text{B}}+\sqrt{{\left(C_{1/1}^{l,\text{F}/\text{B}}\right)}^{2}+{\left(C_{2/2}^{l,\text{F}/\text{B}}\right)}^{2}}\text{cos}\left(\delta +\phi_{++}^{l,\text{F}/\text{B}}\right)\right\}\text{,}$$

(5b) $$T_{--}^{l,\text{F}/\text{B}}=\frac{1}{2}\left\{A_{2/2}^{l,\text{F}/\text{B}}+\sqrt{{\left(C_{3/3}^{l,\text{F}/\text{B}}\right)}^{2}+{\left(C_{4/4}^{l,\text{F}/\text{B}}\right)}^{2}}\text{cos}\left(\delta +\phi_{--}^{l,\text{F}/\text{B}}\right)\right\}\text{,}$$

(5c) $$T_{+-}^{l,\text{F}/\text{B}}=\frac{1}{2}\left\{A_{1/2}^{l,\text{F}/\text{B}}+\sqrt{{\left(C_{1/3}^{l,\text{F}/\text{B}}\right)}^{2}+{\left(C_{2/4}^{l,\text{F}/\text{B}}\right)}^{2}}\text{cos}\left(\delta +\phi_{+-}^{l,\text{F}/\text{B}}\right)\right\}\text{,}$$

(5d) $$T_{-+}^{l,\text{F}/\text{B}}=\frac{1}{2}\left\{A_{2/1}^{l,\text{F}/\text{B}}+\sqrt{{\left(C_{3/1}^{l,\text{F}/\text{B}}\right)}^{2}+{\left(C_{4/2}^{l,\text{F}/\text{B}}\right)}^{2}}\text{cos}\left(\delta +\phi_{-+}^{l,\text{F}/\text{B}}\right)\right\}\text{,}$$

where $A_{p/p^{\prime }}^{l,\text{F}/\text{B}}$ for *p*/*p*′ = 1–2 and $C_{q/q^{\prime }}^{l,\text{F}/\text{B}}$ for *q*/*q*′ = 1–4, for forward *p*, *q* and backward *p*′, *q*′ incidence, are slowly varying functions of the transmission coefficients $t_{ij}^{l,\text{F}/\text{B}}$ and the base angle *α*/−*α*, and

(6a) $$\phi_{++}^{l,\text{F}}=\phi_{+-}^{l,\text{F}}\pm \pi ={\text{tan}}^{-1}\frac{2\left|t_{++}^{l,\text{F}}\right|\left|t_{+-}^{l,\text{F}}\right|\text{sin}\left[\text{arg}\left(t_{+-}^{l,\text{F}}\right)-\text{arg}\left(t_{++}^{l,\text{F}}\right)-2\alpha \right]}{{\left|t_{++}^{l,\text{F}}\right|}^{2}-{\left|t_{+-}^{l,\text{F}}\right|}^{2}}\text{,}$$

(6b) $$\phi_{--}^{l,\text{F}}=\phi_{-+}^{l,\text{F}}\pm \pi ={\text{tan}}^{-1}\frac{2\left|t_{--}^{l,\text{F}}\right|\left|t_{-+}^{l,\text{F}}\right|\text{sin}\left[\text{arg}\left(t_{-+}^{l,\text{F}}\right)-\text{arg}\left(t_{--}^{l,\text{F}}\right)+2\alpha \right]}{{\left|t_{--}^{l,\text{F}}\right|}^{2}-{\left|t_{-+}^{l,\text{F}}\right|}^{2}}\text{,}$$

(6c) $$\phi_{++}^{l,\text{B}}=\phi_{-+}^{l,\text{B}}\pm \pi ={\text{tan}}^{-1}\frac{2\left|t_{++}^{l,\text{B}}\right|\left|t_{-+}^{l,\text{B}}\right|\text{sin}\left[\text{arg}\left(t_{-+}^{l,\text{B}}\right)-\text{arg}\left(t_{++}^{l,\text{B}}\right)-2\alpha \right]}{{\left|t_{++}^{l,\text{B}}\right|}^{2}-{\left|t_{-+}^{l,\text{B}}\right|}^{2}}\text{,}$$

(6d) $$\phi_{--}^{l,\text{B}}=\phi_{+-}^{l,\text{B}}\pm \pi ={\text{tan}}^{-1}\frac{2\left|t_{--}^{l,\text{B}}\right|\left|t_{+-}^{l,\text{B}}\right|\text{sin}\left[\text{arg}\left(t_{+-}^{l,\text{B}}\right)-\text{arg}\left(t_{--}^{l,\text{B}}\right)+2\alpha \right]}{{\left|t_{--}^{l,\text{B}}\right|}^{2}-{\left|t_{+-}^{l,\text{B}}\right|}^{2}}\text{,}$$

are the transmission phases. Note that for unitary transfer matrix $t^{l,\text{F}/\text{B}}$ Eqs. (4)–(6) reduce to those of a single sapphire slab.

It is clear from Eq. (5) that the transmittance for the Au sawtooth metasurface-sapphire system exhibits BI oscillations. However, as we are interested in the relative measurements to eliminate systematic errors and the numerical aperture (NA) effects of the optical system [44], the transmission phase for the metasurface-sapphire system is better characterized by the relative phase w.r.t. that of the sapphire slab, i.e.

(7) $$\Delta \phi_{ij}^{l,\text{F}/\text{B}}=\phi_{ij}^{l,\text{F}/\text{B}}-\phi_{ij}^{\text{Sa}}\text{.}$$

Furthermore, according to the Lorentz Reciprocity Lemma theorem [28], $t_{ii}^{l,\text{F}}=t_{ii}^{l,\text{B}}$ for i=+/− and $t_{ij}^{l,\text{F}}=t_{ji}^{l,\text{B}}$ for i≠j=+/− . Thus, the relative transmission phase for the backward incidence can be expressed in terms of the forward transmission coefficients, leading to:

(8a) $$\Delta \phi_{++}^{l,F}\left(\alpha \right)=\Delta \phi_{+-}^{l,F}\left(\alpha \right)=\Delta \phi_{++}^{l,B}\left(-\alpha \right)=\Delta \phi_{-+}^{l,B}\left(-\alpha \right)\text{,}$$

(8b) $$\Delta \phi_{--}^{l,F}\left(\alpha \right)=\Delta \phi_{-+}^{l,F}\left(\alpha \right)=\Delta \phi_{--}^{l,B}\left(-\alpha \right)=\Delta \phi_{+-}^{l,B}\left(-\alpha \right)\;\text{.}$$

Moreover, as the “atoms” **N** and **И** for our metasurfaces are mirror images of each other and 2D chiral [45], the transmission coefficients of the planar **N**- and **И**-type Au sawtooth metasurfaces satisfy $t_{ii}^{N}=t_{jj}^{N^{-1}}$ and $t_{ij}^{N}=t_{ji}^{N^{-1}}$ for i≠j=+/− . (Note that the base angle α for the planar **N**-/**И**-type sawtooth metasurface under reflection along the $\hat{y}$ -axis will be −α for the **И**-/**N**-type sawtooth metasurface.) Thus, by replacing α by −α for the **И**-type Au sawtooth metasurface, the relative transmission phase for the Au sawtooth metasurface-sapphire system can be further expressed as:

(9a) $$\Delta \phi_{++}^{N,\text{F}}\left(\alpha \right)=\Delta \phi_{+-}^{N,\text{F}}\left(\alpha \right)=\Delta \phi_{--}^{N^{-1},\text{F}}\left(-\alpha \right)=\Delta \phi_{-+}^{N^{-1},\text{F}}\left(-\alpha \right)\text{,}$$

(9b) $$\Delta \phi_{--}^{N,\text{F}}\left(\alpha \right)=\Delta \phi_{-+}^{N,\text{F}}\left(\alpha \right)=\Delta \phi_{++}^{N^{-1},\text{F}}\left(-\alpha \right)=\Delta \phi_{+-}^{N^{-1},\text{F}}\left(-\alpha \right)\text{,}$$

(9c) $$\Delta \phi_{++}^{N,\text{B}}\left(-\alpha \right)=\Delta \phi_{-+}^{N,\text{B}}\left(-\alpha \right)=\Delta \phi_{--}^{N^{-1},\text{B}}\left(\alpha \right)=\Delta \phi_{+-}^{N^{-1},\text{B}}\left(\alpha \right)\text{,}$$

(9d) $$\Delta \phi_{--}^{N,\text{B}}\left(-\alpha \right)=\Delta \phi_{+-}^{N,\text{B}}\left(-\alpha \right)=\Delta \phi_{++}^{N^{-1},\text{B}}\left(\alpha \right)=\Delta \phi_{-+}^{N^{-1},\text{B}}\left(\alpha \right)\text{.}$$

Note that the relative transmission phase defined by Eqs. (8) and (9) for the Au sawtooth metasurface-sapphire system depends only on the transmission coefficients of the metasurface $t_{ij}^{l,\text{F}/\text{B}}$ and the base angle *α* but not on the thickness of the a-cut sapphire slab. Nevertheless, the presence of the a-cut sapphire slab is indispensable as it generates the BI oscillations with peaks and troughs in the transmittance as shown in Eq. (5), enabling the determination of the transmission phase for the different incident and detection polarizations by simply applying the constructive and destructive conditions. Thus, the difference between the transmission phases for the two opposite propagation directions for our **N**- and **И**-type Au sawtooth metasurface-sapphire system can be characterized, following the (traditional) AT notation for chiral metasurface ( $\Delta T_{ij}^{l}\left(\alpha \right)$ = $T_{ij}^{l,\text{F}}\left(\alpha \right)-T_{ij}^{l,\text{B}}\left(-\alpha \right))$ [27], simply by the difference of the relative transmission phase shift w.r.t. that of the sapphire slab, namely:

(10) $$\Delta \Phi_{ij}^{l}\left(\alpha \right)=\Delta \phi_{ij}^{l,\text{F}}\left(\alpha \right)-\Delta \phi_{ij}^{l,\text{B}}\left(-\alpha \right)\text{.}$$

Then, it can be shown from Eq. (8) that:

(11a) $$\Delta \Phi_{ii}^{l}=0$$

for i=+/− and

(11b) $$\Delta \Phi_{ij}^{l}=-\Delta \Phi_{ji}^{l}$$

for i≠j=+/− . Thus, the ATP for the **N**-/**И**-type Au sawtooth chiral metasurface-sapphire system is zero for the co-polarization but nonzero and antisymmetric for the cross-polarization. Furthermore, it can be shown from Eq. (9) that:

(12) $$\Delta \Phi_{ij}^{N}\left(\alpha \right)=\Delta \Phi_{ji}^{N^{-1}}\left(-\alpha \right)=-\Delta \Phi_{ij}^{N^{-1}}\left(-\alpha \right)$$

for i,j=+/− . Equations (11) and (12) summarize well the rich asymmetry transmission phase properties of the **N**-/**И**-type Au sawtooth metasurface-sapphire system. Importantly, now there is an additional degree of freedom for controlling the ATP of the Au sawtooth metasurface-sapphire system, namely the base angle α as shown in Eq. (6), providing a new avenue to achieve specific phase modulations or controls.

## 3 Experiment

### 3.1 Fabrication of the Au sawtooth nanoarray metasurface

Following the procedures reported earlier [37], our planar Au sawtooth nanoarray metasurface sample was fabricated by using first an e-beam evaporation technique to deposit a 30 nm Au film onto a nonbirefringent substrate and then a focus ion-beam technique to direct-write 50 × 50 μm^2^ sawtooth nanohole-array patterns onto the Au film. Figure 1c and d show, respectively, SEM images of the planar (30 nm thick) **N-** and **И**-type Au sawtooth metasurfaces with 450 nm lattice spacing’s in both the horizontal and vertical directions for the visible range. The insets of Figure 1c and d are enlarged images of the unit cells for the Au sawtooth metasurfaces. Note that the Au sawtooth nanoarrays have a half lattice horizontal shift between alternate rows so that each Au sawtooth nanoarray is disconnected from the above and below neighboring Au sawtooth nanoarrays.

### 3.2 Bidirectional transmission and phase measurements

**Figure 2:**
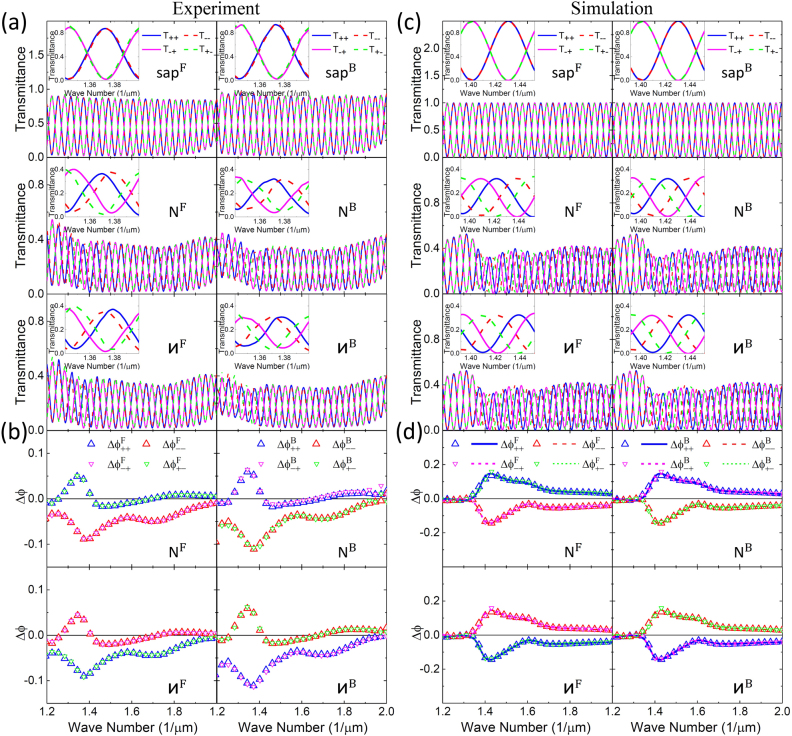
Bidirectional transmittance and transmission phase. (a) and (b) Measurements of transmittance and transmission phase for circularly polarized incidence for forward (left column)/backward (right column) direction of the metasurface systems placed on the top of an *L* = 2 mm a-cut sapphire birefringence crystal slab with base angle *α* = 30°/−30° for the forward and −*α* = −30°/30° for the backward directions. The insets are the transmittance on an expanded scale for about one birefringence interference period. Open triangles in (b) are transmission phase w.r.t. the a-cut sapphire obtained from Eq. (15). (c) and (d) Corresponding transmittance and transmission phase from simulations for the experimental results in (a) and (b). Symbols are transmission phases obtained from the peak shift method using Eq. (15) and curves are calculations using Eq. (7).

Figure 2a shows the transmittance, obtained using the procedures reported earlier [37], of our Au sawtooth metasurface placed on an a-cut sapphire slab, thickness *L* = 2 mm, for the forward (left column) and backward (right column) directions with a base angle *α* = 30°/−30° and −*α* = −30°/30°, respectively. It is clear that the transmittance for the sapphire slab and the **N-**/**И**-type Au sawtooth metasurface all exhibit BI oscillations due to the phase difference δ = $\frac{2\pi }{\lambda }\left(n_{o}-n_{e}\right)L$ between the fast axis (extraordinary $\hat{e}$ -axis) and the slow axis (ordinary $\hat{o}$ -axis) components of the a-cut sapphire slab. In general, the experimental transmission BI oscillations can be represented as [46]: (details about the experimental transmittance and transmission phase can be found in Section 2 of the Supplementary Material.)

(13) $$T_{ij}^{l}=A_{ij}+B_{ij}\text{ cos}\left(\frac{2\pi }{\lambda_{ij}^{l}}\left(n_{\text{o}}-n_{\text{e}}\right)L+\phi_{ij}^{l}\right)\text{,}$$

where $\phi_{ij}^{l}$ is the total transmission phase of the Au sawtooth metasurface-sapphire system; $A_{ij}$ and $B_{ij}$ are slowly varying functions of the wavenumber due to the dispersion of the sapphire and Au sawtooth nanoarrays. Equation (13) exhibits transmission peaks/troughs for constructive/destructive interference satisfying the condition:

(14) $$m=\frac{1}{\lambda_{ij}^{l,m}}\left(n_{\text{o}}-n_{\text{e}}\right)L+\phi_{ij}^{l,m}\text{,}$$

with $\lambda_{ij}^{l,m}$ the peak/trough wavelength of the $m^{\text{th}}$ / ${\left(m+1/2\right)}^{\text{th}}$ interference order. (Note that the transmission phase $\phi_{ij}^{l,m}$ is now normalized by 2*π* and *l* = Sa/N/N^−1^.) It is clear from the insets of Figure 2a that the transmittance for the sapphire slab, corresponding to a unitary $t^{l,m}$ in Eq. (2), $T_{++}^{\text{Sa}}\left(=T_{--}^{\text{Sa}}\right)$ is π out of phase with $T_{+-}^{\text{Sa}}\left(=T_{-+}^{\text{Sa}}\right)$ and does not depend on the propagation direction, in good agreement with Eqs. (5) and (6). (See Section 1 of the Supplementary Material.) However, the transmittance for the chiral metasurfaces, the **N**- and **И**-type Au sawtooth nanoarray gratings, show different shifts w.r.t. that of the sapphire slab. The shift directions for the forward and backward incidence remain the same for co-polarized components while they are opposite for the cross-polarized components. (See the inset transmittance in the second-row/third-row of Figure 2a for the **N**-/**И**-type Au sawtooth metasurfaces.) Importantly, the trends are complementary between the **N**- and **И**-type Au sawtooth metasurfaces as they are mirror images of each other.

To obtain more robust results, we characterize the transmission phase simply by the phase shift w.r.t. that of the sapphire slab to cancel out the systematic errors of the optical system [44]. Thus, the transmission phase of the planar Au sawtooth metasurface can be evaluated at the peaks/troughs (peak shift method) by:

(15) $$\Delta \phi_{ij}^{l,m}=\left(\frac{\frac{1}{\lambda_{ij}^{\text{sa},m}}-\frac{1}{\lambda_{ij}^{l,m}}}{\frac{1}{\lambda_{ij}^{\text{sa},m+1}}-\frac{1}{\lambda_{ij}^{\text{sa},m}}}\right)\text{.}$$

Note that the transmission phase $\Delta \phi_{ij}^{l,m}$ given by Eq. (15) is equal to the relative transmission phase defined in Eq. (7) for the Au sawtooth metasurface.

Figure 2b shows the transmission phase $\Delta \phi_{ij}^{l,m}$ relative to those of the sapphire slab for the **N**-(**И**-)type Au sawtooth metasurface with base angle *α* = 30°(−30°) for the forward and −*α* = −30°(30°) for the backward directions. The relative transmission phases for the **N**- and **И**-type Au sawtooth metasurfaces are complementary as shown in the left column of Figure 2b for the forward incidence, and similarly for the backward incidence in the right column, as the fabricated Au sawtooth nanoarrays are almost mirrored images of each other, verifying the high quality of our samples. It is also clear from the graphs in the first row of Figure 2b that the relative transmission phases for the **N**-type Au sawtooth metasurface are, within the experimental errors, the same for the same transmitted component for the forward incidence, i.e., $\Delta \phi_{ii}^{\text{F}}=\Delta \phi_{ij}^{\text{F}}$ , and also for the same incident component for the backward incidence, i.e., $\Delta \phi_{ii}^{\text{B}}=\Delta \phi_{ji}^{\text{B}}$ , in good agreement with Eq. (8). Furthermore, the transmission phases for the different incident directions are nearly the same for the same co-polarization components and also for the interchanged cross-polarization components, i.e., $\Delta \phi_{ii}^{\text{F}}\left(\alpha \right)=\Delta \phi_{ii}^{\text{B}}$ (−α) for i=+/− , and $\Delta \phi_{ij}^{\text{F}}\left(\alpha \right)=\Delta \phi_{ji}^{\text{B}}\left(-\alpha \right)$ for i≠j=+/− , as shown in the first row of Figure 2b, in good agreement with Eq. (8). The above trends are also observed for the **И**-type Au sawtooth metasurface as shown in the second row of Figure 2b. Moreover, the relative transmission phase exhibits peak/trough with values as high as ∼0.1, e.g., at wavenumber around ∼1.37 (1/μm). Importantly, the value of the relative transmission phase, as well as the corresponding peak/trough wavenumber, can be tuned by changing the base angle *α*, enabling the controllability of the metasurface-sapphire system for asymmetric transmission applications. (Detailed discussion about the ATP of Au sawtooth metasurface-sapphire system can be found in Section 5 of the Supplementary Material.)

### 3.3 Asymmetric transmission phase

**Figure 3:**
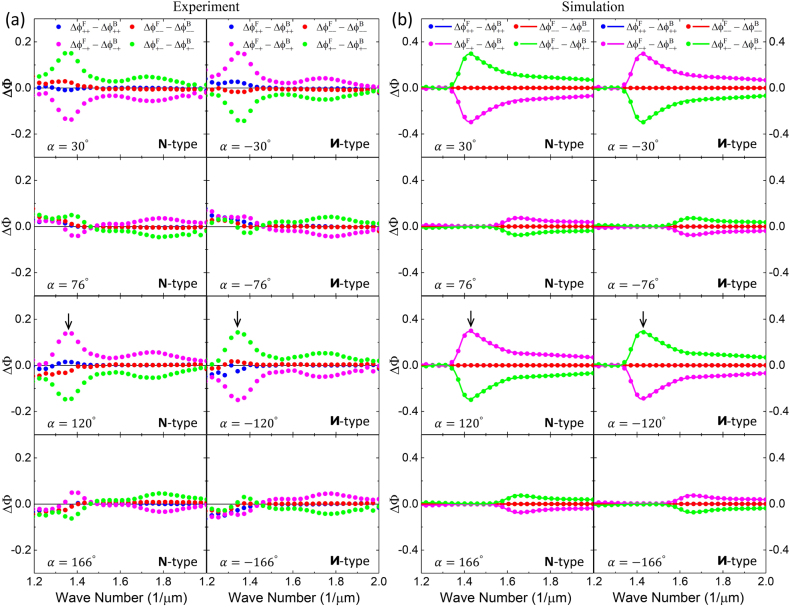
Asymmetric transmission phase. (a) Experimental results of ATP for the **N**- and И -type Au sawtooth nanoarray metasurface-sapphire systems for various base angles α chosen for extreme responses at wavenumber 1/*λ* ∼ 1.37 μm^−1^. (b) Corresponding ATP from simulations. Solid dots are obtained from the peak shift method using Eqs. (10) and (15) and curves are calculations using Eqs. (7) and (10). Black arrows indicate the local maxima.

**Figure 4:**
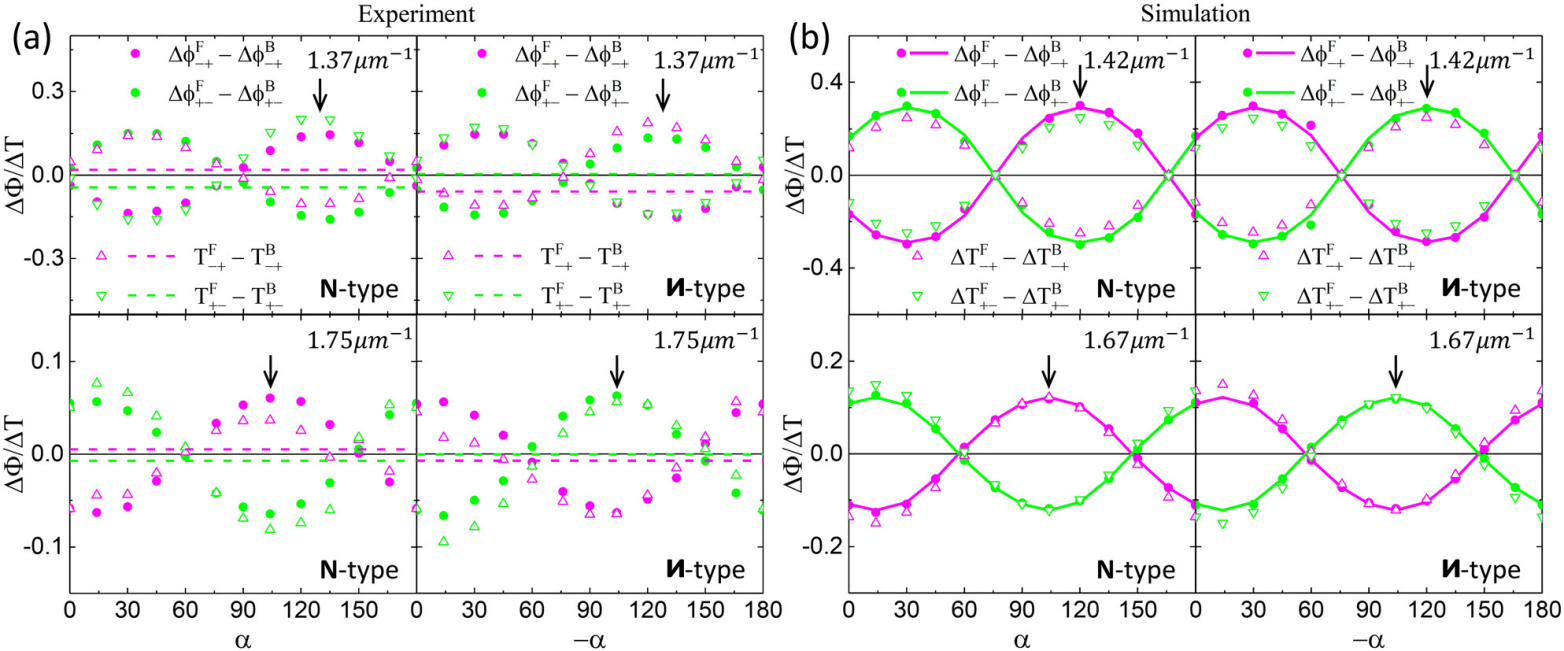
Controllable asymmetric transmission and transmission phase. (a) Results of ATP (solid dots) at wavenumbers 1.37 μm^−1^ and 1.75 μm^−1^ and AT (open triangles) at corresponding wavenumbers for the **N**- and И -type Au sawtooth metasurface-sapphire system with the controllable base angle *α*. Dashed lines are AT for the Au sawtooth metasurface without the sapphire slab. (b) Corresponding ATP (solid dots and curves) and transmission (open triangles) from simulations at wavenumbers 1.42 μm^−1^ and 1.67 μm^−1^. Dots are obtained from the peak shift method using Eqs. (10) and (15) and curves are calculations using Eqs. (7) and (10). Black arrows indicate the local maxima.

Figure 3 shows the experimental results of the bidirectional transmission phase difference, defined in Eq. (10) as the ATP $\Delta \Phi_{ij}^{l}\left(\alpha \right)$ of the **N**- and **И**-type Au sawtooth nanoarray metasurface-sapphire system for various base angles α chosen for extreme responses at wavenumber 1/*λ* ∼ 1.37 μm^−1^. The ATP is almost zero within the experimental errors for the co-polarization components (blue and red dots in Figure 3a), in good agreement with Eq. (11a). Importantly, the ATP is nearly antisymmetric for the cross-polarization components (green and magenta dots in Figure 3a) also in good agreement with Eq. (11b). Furthermore, comparing the results for the **N**- and **И**-type Au sawtooth nanoarray metasurface-sapphire systems, $\Delta \Phi_{ij}^{N}\left(\alpha \right)∼\Delta \Phi_{ji}^{N^{-1}}\left(-\alpha \right)∼-\Delta \Phi_{ij}^{N^{-1}}\left(-\alpha \right)$ for i≠j=+/− , in good agreement with Eq. (12). In addition to the symmetry properties, the ATP also exhibits extremum at wavenumber 1/*λ* ∼ 1.37 μm^−1^ as indicated by the black arrows. (Note that the local extremum at 1/*λ* ∼ 1.75 μm^−1^ occurs at different base angles as described in Figure 10 of the Supplementary Material.) The 1/*λ* ∼ 1.37 μm^−1^ and ∼1.75 μm^−1^ extrema, with amplitudes of ∼0.15 and ∼0.06, respectively, correspond to the different resonances of the Au sawtooth nanoarrays. (See Figure 5 about the resonances of Au sawtooth metasurface in Section 4 of the Supplementary Material.) Importantly, the ATP at these two resonances can be controlled in an oscillatory manner by varying the base angle as shown in Figure 4a. For example at 1/*λ* ∼ 1.37 μm^−1^, the ATP for both the **N**- and **И**-type Au sawtooth nanoarray metasurface-sapphire systems has an extremum around *α* ∼ 35°, becomes zero around *α* ∼ 80°, and then reverses the sign around *α* ∼ 125°. The same trend is observed for 1/*λ* ∼ 1.75 μm^−1^ with a peak around *α* ∼ 15°, zero around *α* ∼ 60°, and a trough around *α* ∼ 105°. The base angle dependence for the ATP is inherited from the *α* dependence in Eq. (6) with a *π* periodicity.

The controllability of the ATP for the **N**-/**И**-type Au sawtooth metasurface-sapphire system can also apply to the AT ( $\Delta T_{ij}^{l}\left(\alpha \right)$ = $T_{ij}^{l,\text{F}}\left(\alpha \right)-T_{ij}^{l,\text{B}}\left(-\alpha \right))$ obtained by simply taking the difference between the forward and backward transmittance of the metasurface-sapphire systems for different base angles. However, a simple difference of the forward and backward transmittance as shown in Figure 2a will give undesirable results as the substrate and/or the sapphire slab, with asymmetric losses, is breaking the symmetry of the metasurface-sapphire system. The substrate effect can be rectified/reduced by adding matching substrates to the system [38] or by re-normalizing the backward transmittance by that of the forward transmittance as illustrated in Figures S1 and S3. (Detailed discussion about the transmittance and AT of Au sawtooth metasurface-sapphire system can be found in Section 3 of the Supplementary Material.) The AT, after renormalization, shows oscillatory responses similar to that of the ATP as shown in Figure 4a for the **N**-/**И**-type Au sawtooth metasurface-sapphire system. (See Figure S11 about the AT of Au sawtooth metasurface-sapphire system in Section 6 of the Supplementary Material.) The AT (open triangles), exhibits responses with comparable values as the ATP (solid dots). However, it is more susceptible to experimental errors as seen in the slightly nonsymmetric behavior as shown in Figure 4a. Displayed also in Figure 4a are the traditional AT (magenta and green dashed lines) for the **N**-/**И**-type Au sawtooth metasurface alone obtained from Figure 5 of the Supplementary Material at 1/*λ* ∼ 1.36 μm^−1^ and ∼1.74 μm^−1^ extrema. Note that the traditional AT of the metasurface alone is constant independent of the base angle *α* as there is no tuning parameter. It is clear that the AT for the metasurface-sapphire system, overall, is much larger, and more importantly, can be tuned by controlling the base angle *α*, similar to that of the ATP.

To further elucidate our approach on other systems, we fabricate achiral planar and complementary double-layer plasmonic metasurfaces and obtain the AT and ATP as described in Section 7 of the Supplementary Material. The results show similar tunable behaviors as the sawtooth metasurfaces above with the add-on sapphire slab despite the original “atoms” of the metasurfaces being achiral. Moreover, the response for the ATP can be much larger than that of the AT for the complementary double-layer metasurface. These results demonstrate the advantage of using the add-on sapphire slab to control the chiral properties of metasurfaces/metamaterials. (Detailed discussion about the application of our approach to achiral metasurfaces can be found in Section 7 of the Supplementary Material.)

To conclude, the ATP is more robust than the AT as the phase is not sensitive to fluctuations of the signal amplitude while the transmittance is directly affected. Thus the ATP serves as a good alternative, if not complementing the AT, for the characterization of asymmetric transport of chiral materials. Furthermore, our results demonstrate well the tunability advantage of adding the sapphire slab to metasurfaces. Note that the transmission efficiency of the metasurface-sapphire system could be improved by replacing the Au with dielectric materials for the metasurface [40, 41]. Moreover, the sapphire slab is applicable in the optical wavelengths as it is transparent from the ultraviolet to the infrared spectrum. Furthermore, the uniaxial birefringent sapphire slab used in this work can be replaced by a biaxial birefringent material or any substrate inscribed with various anisotropies to further increase the degrees of freedom in wave controls and manipulations.

## 4 Simulation of Au sawtooth metasurface-sapphire system

### 4.1 Full-wave simulation

To support our approach in using the BI approach for characterizing ATP with tunable responses in chiral metasurface we perform full-wave simulations to cross-check our experimental results. Instead of simulating the whole Au sawtooth metasurface-sapphire chiral system, we follow the approach used for the transmission phase as reported recently [37]. Firstly, the Jones matrix $t^{l}$ of the free-standing planar Au sawtooth metasurface for the forward and backward incidence are obtained for base angle *α* = 0 by using a finite-integration-technique simulator from CST Microwave Studio with periodic boundary conditions and unit cells constructed to resemble those of the experiments as shown in Figure 1c and d. Then we use Eq. (5) to calculate the transmittance in the forward and backward directions for the Au sawtooth metasurface-sapphire system for various base angles *α*. Note that the transmittance is now dependent on the base angle of the sawtooth nanoarray w.r.t. the optical axis of the sapphire slab. Finally, we calculate the transmission phase, and hence the ATP, using the peak shift method given by Eqs. (10) and (15) for the peaks/troughs of the transmission BI oscillations and also directly by using Eqs. (7) and (10).

### 4.2 Simulation results

Figure 2c and d show the transmittance and transmission phase of the **N**- and **И**-type Au sawtooth metasurface-sapphire systems for the forward and backward incidence with base angles *α* corresponding to those of the experiments as shown in Figure 2a and b. The overall transmittance, also in expanded scales in the insets, resembles the experimental results very well. Similarly, the transmission phase (relative to that of the sapphire) exhibits very similar responses as the experiment.

Figure 3b shows the corresponding simulation results of the ATP for the experimental results for various base angles *α* as shown in Figure 3a. The simulations agree very well with the experiments except that the local maximum at 1/*λ* ∼ 1.37 μm^−1^ (indicated by the black arrows) observed in the experiment is now shifted to 1/*λ* ∼ 1.42 μm^−1^ due to the substrate effect and also the physical parameters (dimensions of the Au sawtooth nanoarrays and dielectric constants of the materials) used in the simulations may not match those of the experiment perfectly. Note that the simulation results obtained by the peak shift method using Eqs. (10) and (15) (symbols) are the same as those obtained directly from calculations using Eqs. (7) and (10) (solid curves), supporting the use of the peak shift method for the experiments. Note also that the second local maximum at 1/*λ* ∼ 1.75 μm^−1^ observed in the experiment as shown in Figure 3a is not as obvious in the simulations for the chosen base angles. However, it is noticeable at the base angle *α* ∼ 14°/104° with a shift to 1/*λ* ∼ 1.67 μm^−1^ as shown in Figure 10 of the Supplementary Material.

Figure 4b summarizes the simulated results for the ATP of the **N**- and **И**-type Au sawtooth metasurface-sapphire systems at the local maxima, 1/*λ* ∼ 1.42 μm^−1^ and 1/*λ* ∼ 1.67 μm^−1^, as a function of the base angle. The oscillatory dependence agrees very well with the experimental results shown in Figure 4a despite that the angles for the local maxima are slightly shifted and also the magnitudes are larger. Overall, the simulations confirm our experimental results and support the use of the sapphire slab as a tunable element to control the ATP, as well as the traditional AT.

## 5 Conclusions

We propose a novel approach in the characterization of planar chiral metasurface with a controllable asymmetric transmission phase using a simple interference technique by adding a birefringent crystal, here a uniaxial a-cut sapphire slab, to the metasurface. To demonstrate our approach, we measure the ATP of **N**-/**И**-type Au sawtooth nanoarray chiral metasurface placed on the sapphire slab as a function of the controllable base angle between the nanoarray of the metasurface and the optical axis of the sapphire slab. To cross-check our approach, we also perform full-wave simulations of our Au sawtooth metasurface-sapphire system and obtain good agreement with the experiments. The tunability approach by adding a birefringent substrate can also be applied to the traditional AT. Importantly, the metasurface does not have to be fabricated directly on the birefringent substrate and the add-on birefringent plate can be incorporated as a lens or a filter for a measuring device. Thus our approach is flexible and nondestructive, offering great potentials in optical communication, imaging, and remote sensing applications.

## Supplementary material

The supplementary materials are available for some supporting information. Section 1 gives the Jones matrix for the metasurface-sapphire system; Section 2 shows the experimental transmittance and transmission phase; Section 3 presents the transmittance and asymmetric transmission of Au sawtooth metasurface-sapphire system; Section 4 explains the resonances of Au sawtooth metasurface; Section 5 provides more results for the asymmetric transmission phase of Au sawtooth metasurface-sapphire system; Section 6 demonstrates the asymmetric transmission of Au sawtooth metasurface-sapphire system; Section 7 shows applications to achiral metasurfaces.

## Supplementary Material

Supplementary Material Details

Supplementary Material Details
