# Supplementary material for: Controlling asymmetric transmission phase in planar chiral metasurfaces
Source: Nanophotonics. 2021 Dec 22;11(3):495–505. doi: 10.1515/nanoph-2021-0558 (PMC11501898; doi:10.1515/nanoph-2021-0558)
Supplement: Supplementary file 1 — Supplementary Material Details [file j_nanoph-2021-0558_suppl_001.docx]

Supplementary Material for the manuscript:

**Controlling asymmetric transmission phase in planar chiral metasurfaces**

Ranran Zhang,^a,b^ Qiuling Zhao,^a,*^ Xia Wang,^a,*^ Kai Ming Lau,^b^ Tsz Kit Yung,^b^ Jensen Li,^b^ Wing Yim Tam^a,b,*^

^a^Optoelectronic Materials and Technologies Engineering Laboratory, Shandong, Physics Department, QingDao University of Science and Technology, Shandong, China

^b^Department of Physics and William Mong Institute of Nano Science and Technology, Hong Kong University of Science and Technology, Clear Water Bay, Kowloon, Hong Kong, China

ng, China

1. **Jones matrix for the metasurface-sapphire system**

We use the Jones matrix approach to model the propagation of waves (propagating in the positive $\hat{z}$-axis direction) for our metasurface-sapphire system [1]. For Jones representation, the normal incident transfer matrix in circular polarization bases for an a-cut birefringent sapphire slab with the optical axis chosen as the reference $\hat{x}$-axis can be written as [2]:

$\boldsymbol{t}^{Sa}=\left( \begin{matrix} t_{++}^{Sa} & t_{+-}^{Sa} \\ t_{-+}^{Sa} & t_{--}^{Sa} \end{matrix} \right)=\left( \begin{matrix} \cos(\frac{\delta}{2}) & isin(\frac{\delta}{2}) \\ isin(\frac{\delta}{2}) & \cos(\frac{\delta}{2}) \end{matrix} \right)$ , (S1)

where $\delta$ ***=*** $\frac{2\pi}{\lambda}\left( n_{o}-n_{e} \right)L$ for *L* the thickness of the sapphire. We choose +/- to denote the right/left-handed circularly polarization (RCP/LCP). The subscript indexes, *i* and *j* (for $i,j=+/-)$, of $t_{ij}^{Sa}$ are the outgoing and incoming rays, respectively. Thus, the transmission coefficients for RCP $\left( \begin{matrix} 1 \\ 0 \end{matrix} \right)$ and LCP $\left( \begin{matrix} 0 \\ 1 \end{matrix} \right)$ incidence can be expressed as:

$\left( \begin{matrix} E_{++}^{Sa} \\ E_{-+}^{Sa} \end{matrix} \right)=\boldsymbol{t}^{Sa}\left( \begin{matrix} 1 \\ 0 \end{matrix} \right)=\left( \begin{matrix} \cos(\frac{\delta}{2}) & isin(\frac{\delta}{2}) \\ isin(\frac{\delta}{2}) & \cos(\frac{\delta}{2}) \end{matrix} \right)\left( \begin{matrix} 1 \\ 0 \end{matrix} \right)=\left( \begin{matrix} \cos(\frac{\delta}{2}) \\ isin(\frac{\delta}{2}) \end{matrix} \right)$ (S2a)

and

$\left( \begin{matrix} E_{+-}^{Sa} \\ E_{--}^{Sa} \end{matrix} \right)=\boldsymbol{t}^{Sa}\left( \begin{matrix} 0 \\ 1 \end{matrix} \right)=\left( \begin{matrix} \cos(\frac{\delta}{2}) & isin(\frac{\delta}{2}) \\ isin(\frac{\delta}{2}) & \cos(\frac{\delta}{2}) \end{matrix} \right)\left( \begin{matrix} 0 \\ 1 \end{matrix} \right)=\left( \begin{matrix} isin(\frac{\delta}{2}) \\ \cos(\frac{\delta}{2}) \end{matrix} \right)$ , (S2b)

respectively.

The transmittance through the sapphire plate can then be written as:

$T_{++}^{Sa}=E_{++}^{Sa}{\cdot E}_{++}^{Sa*}={[\cos(\frac{\delta}{2})]}^{2} = \frac{1}{2}(1+\cos\delta)= \frac{1}{2}(1+\cos\left( \delta+\phi_{++}^{Sa} \right))$, (S3a)

$T_{--}^{Sa}=E_{--}^{Sa}{\cdot E}_{--}^{Sa*}= {[\cos(\frac{\delta}{2})]}^{2}=\frac{1}{2} (1+\cos\delta) =\frac{1}{2} (1+\cos(\delta+\phi_{--}^{Sa})$, (S3b)

$T_{+-}^{Sa}=E_{+-}^{Sa}\cdot E_{+-}^{Sa*}= \left[ \sin\left( \frac{\delta}{2} \right) \right]^{2}= \frac{1}{2}(1-\cos\delta)= \frac{1}{2}(1+\cos(\delta\pm\pi))=\frac{1}{2}(1+\cos\left( \delta+\phi_{+-}^{Sa} \right))$, (S3c)

$T_{-+}^{Sa}=E_{-+}^{Sa}\cdot E_{-+}^{Sa*}= \left[ \sin\left( \frac{\delta}{2} \right) \right]^{2}= \frac{1}{2}(1-\cos\delta)=\frac{1}{2}(1+\cos(\delta\pm\pi))=\frac{1}{2}(1+\cos\left( \delta+\phi_{-+}^{Sa} \right))$. (S3d)

Clearly, $T_{++}^{Sa}=T_{--}^{Sa}$ is *π* out of phase with $T_{+-}^{Sa}=T_{-+}^{Sa}$, and the transmission phase of the sapphire slab can be written as:

$\phi_{++}^{Sa}=\phi_{--}^{Sa}=0$ , (S4a)

and

$\phi_{-+}^{Sa}=\phi_{+-}^{Sa}=\pm\pi$. (S4b)

Note that as $\delta$ varies with a period inversely proportional to *L* all transmittance exhibits birefringent interference (BI) oscillations similar to those oscillations due to the Fabry-Perot interference [3]. Note also that the transmittance of the sapphire slab is independent of the incident (forward or backward) direction.

Using the same approach, the transfer matrix in the Jones representation for a metasurface at normal incidence can be written in circular polarization bases as [4]:

$\boldsymbol{t}^{l,F}(\alpha)=\left( \begin{matrix} t_{++}^{l,F} & e^{-i2\alpha}t_{+-}^{l,F} \\ e^{i2\alpha}t_{-+}^{l,F} & t_{--}^{l,F} \end{matrix} \right)$ (S5a)

for the forward (F) incidence and

$\boldsymbol{t}^{l,B}(-\alpha)=\left( \begin{matrix} t_{++}^{l,B} & e^{i2\alpha}t_{+-}^{l,B} \\ e^{-i2\alpha}t_{-+}^{l,B} & t_{--}^{l,B} \end{matrix} \right)$ (S5b)

for the backward (B) incidence, where *l* = *N*/*N^-1^* denotes our planar **N**-/**и**-type chiral metasurface. *α /-α* is the forward/backward base angle (positive for anti-clockwise and negative for clockwise) between the lattice/“atom” orientation of the metasurface and the $\hat{x}$-axis, here it is the optical axis of the a-cut sapphire slab. $t_{ij}^{l,F/B}= \left| t_{ij}^{l,F/B} \right|e^{iArg(t_{ij}^{l,F/B})}$ is the transmission coefficient for $i,j=+/-$.

Then, the total transfer matrix for the metasurface-sapphire system for the forward ($\boldsymbol{E}^{l,F}$) and backward ($\boldsymbol{E}^{l,B}$) incidence can be written in circular polarization base as:

$\boldsymbol{E}^{l,F}(\alpha)=\left( \begin{matrix} E_{++}^{l,F} & E_{+-}^{l,F} \\ E_{-+}^{l,F} & E_{--}^{l,F} \end{matrix} \right)= \boldsymbol{t}^{l,F}(\alpha)\boldsymbol{t}^{Sa}=\left( \begin{matrix} t_{++}^{l,F} & e^{-i2\alpha}t_{+-}^{l,F} \\ e^{i2\alpha}t_{-+}^{l,F} & t_{--}^{l,F} \end{matrix} \right)\left( \begin{matrix} \cos(\frac{\delta}{2}) & isin(\frac{\delta}{2}) \\ isin(\frac{\delta}{2}) & \cos(\frac{\delta}{2}) \end{matrix} \right)$ (S6a)

and

$\boldsymbol{E}^{l,B}(-\alpha)=\left( \begin{matrix} E_{++}^{l,B} & E_{+-}^{l,B} \\ E_{-+}^{l,B} & E_{--}^{l,B} \end{matrix} \right)= \boldsymbol{t}^{Sa}\boldsymbol{t}^{l,B}(-\alpha)=\left( \begin{matrix} \cos(\frac{\delta}{2}) & isin(\frac{\delta}{2}) \\ isin(\frac{\delta}{2}) & \cos(\frac{\delta}{2}) \end{matrix} \right) \left( \begin{matrix} t_{++}^{l,B} & e^{i2\alpha}t_{+-}^{l,B} \\ e^{-i2\alpha}t_{-+}^{l,B} & t_{--}^{l,B} \end{matrix} \right)$. (S6b)

(Note that for unitary $\boldsymbol{t}^{l,F/B}$ Eq. (S6) reduces to that of the sapphire slab.) Furthermore, the forward transmittance can then be written as:

$$T_{++}^{l,F}=E_{++}^{l,F}{\cdot E}_{++}^{l,F*}$$

$$=\frac{1}{2}\left\{ \left| t_{++}^{l,F} \right|^{2}+\left| t_{+-}^{l,F} \right|^{2}+\left( \left| t_{++}^{l.F} \right|^{2}-\left| t_{+-}^{l,F} \right|^{2} \right)\cos\delta-2\left| t_{++}^{l,F} \right|\left| t_{+-}^{l,F} \right|\sin[arg\left( t_{+-}^{l,F} \right)-arg\left( t_{++}^{l,F} \right)-2\alpha]\sin\delta\right\}$$

$=\frac{1}{2}\left\{ A_{1}^{l,F}+\sqrt{{(C_{1}^{l,F})}^{2}+{(C_{2}^{l,F})}^{2}}\cos(\delta+\phi_{++}^{l,F}) \right\}$ , (S7a)

$$T_{--}^{l,F}=E_{--}^{l,F}{\cdot E}_{--}^{l,F*}$$

$$=\frac{1}{2}\left\{ \left| t_{--}^{l,F} \right|^{2}+\left| t_{-+}^{l,F} \right|^{2}+\left( \left| t_{--}^{l,F} \right|^{2}-\left| t_{-+}^{l,F} \right|^{2} \right)\cos\delta-2\left| t_{--}^{l,F} \right|\left| t_{-+}^{l,F} \right|\sin[arg\left( t_{-+}^{l,F} \right)-arg\left( t_{--}^{l,F} \right)+2\alpha]\sin\delta\right\}$$

$=\frac{1}{2}\left\{ A_{2}^{l,F}+\sqrt{{(C_{3}^{l,F})}^{2}+{(C_{4}^{l,F})}^{2}}\cos(\delta+\phi_{--}^{l,F}) \right\}$ , (S7b)

$$T_{+-}^{l,F}=E_{+-}^{l,F}{\cdot E}_{+-}^{l,F*}$$

$$=\frac{1}{2}\left\{ \left| t_{++}^{l,F} \right|^{2}+\left| t_{+-}^{l,F} \right|^{2}-\left( \left| t_{++}^{l,F} \right|^{2}-\left| t_{+-}^{l,F} \right|^{2} \right)\cos\delta+2\left| t_{++}^{l,F} \right|\left| t_{+-}^{l,F} \right|\sin[arg\left( t_{+-}^{l,F} \right)-arg\left( t_{++}^{l,F} \right)-2\alpha]\sin\delta\right\}$$

$=\frac{1}{2}\left\{ A_{1}^{l,F}+\sqrt{{(C_{1}^{l,F})}^{2}+{(C_{2}^{l,F})}^{2}}\cos(\delta+\phi_{+-}^{l,F}) \right\}$, (S7c)

$$T_{-+}^{l,F}=E_{-+}^{l,F}{\cdot E}_{-+}^{l,F*}$$

$$=\frac{1}{2}\left\{ \left| t_{--}^{l,F} \right|^{2}+\left| t_{-+}^{l,F} \right|^{2}-\left( \left| t_{--}^{l,F} \right|^{2}-\left| t_{-+}^{l,F} \right|^{2} \right)\cos\delta+2\left| t_{--}^{l,F} \right|\left| t_{-+}^{l,F} \right|\sin[arg\left( t_{-+}^{l,F} \right)-arg\left( t_{--}^{l,F} \right)+2\alpha]\sin\delta\right\}$$

$=\frac{1}{2}\left\{ A_{2}^{l,F}+\sqrt{{(C_{3}^{l,F})}^{2}+{(C_{4}^{l,F})}^{2}}\cos(\delta+\phi_{-+}^{l,F}) \right\}$, (S7d)

where

$\left\{ \begin{aligned} A_{1}^{l,F}=\left| t_{++}^{l,F} \right|^{2}+\left| t_{+-}^{l,F} \right|^{2} \\ A_{2}^{l,F}=\left| t_{--}^{l,F} \right|^{2}+\left| t_{-+}^{l,F} \right|^{2} \\ C_{1}^{l,F}=\left| t_{++}^{l,F} \right|^{2}-\left| t_{+-}^{l,F} \right|^{2} \\ C_{2}^{l,F}=2\left| t_{++}^{l,F} \right|\left| t_{+-}^{l,F} \right|\sin[arg\left( t_{+-}^{l,F} \right)-arg\left( t_{++}^{l,F} \right)-2\alpha] \\ C_{3}^{l,F}=\left| t_{--}^{l,F} \right|^{2}-\left| t_{-+}^{l,F} \right|^{2} \\ C_{4}^{l,F}=2\left| t_{--}^{l,F} \right|\left| t_{-+}^{l,F} \right|\sin[arg\left( t_{-+}^{l,F} \right)-arg\left( t_{--}^{l,F} \right)+2\alpha] \end{aligned} \right.$ (S8)

and

$\phi_{++}^{l,F}=\phi_{+-}^{l,F}\pm\pi= {tan}^{-1} \frac{2\left| t_{++}^{l,F} \right|\left| t_{+-}^{l,F} \right|\sin\left[ arg\left( t_{+-}^{l,F} \right)-arg\left( t_{++}^{l,F} \right)-2\alpha\right]}{\left| t_{++}^{l,F} \right|^{2}-\left| t_{+-}^{l,F} \right|^{2}}$ , (S9a)

$\phi_{--}^{l,F}=\phi_{-+}^{l,F}\pm\pi= {tan}^{-1} \frac{2\left| t_{--}^{l,F} \right|\left| t_{-+}^{l,F} \right|\sin\left[ arg\left( t_{-+}^{l,F} \right)-arg\left( t_{--}^{l,F} \right)+2\alpha\right]}{\left| t_{--}^{l,F} \right|^{2}-\left| t_{-+}^{l,F} \right|^{2}}$ . (S9b)

Similarly, the backward transmittance can be written as:

$$T_{++}^{l,B}=E_{++}^{l,B}{\cdot E}_{++}^{l,B*}$$

$$=\frac{1}{2}\left\{ \left| t_{++}^{l,B} \right|^{2}+\left| t_{-+}^{l,B} \right|^{2}+\left( \left| t_{++}^{l,B} \right|^{2}-\left| t_{-+}^{l,B} \right|^{2} \right)\cos\delta-2\left| t_{++}^{l,B} \right|\left| t_{-+}^{l,B} \right|\sin[arg\left( t_{-+}^{l,B} \right)-arg\left( t_{++}^{l,B} \right)-2\alpha]\sin\delta\right\}$$

$=\frac{1}{2}\left\{ A_{1}^{l,B}+\sqrt{{(C_{1}^{l,B})}^{2}+{(C_{2}^{l,B})}^{2}}\cos(\delta+\phi_{++}^{l,B}) \right\}$ , (S10a)

$$T_{--}^{l,B}=E_{--}^{l,B}{\cdot E}_{--}^{l,B*}$$

$$=\frac{1}{2}\left\{ \left| t_{--}^{l,B} \right|^{2}+\left| t_{+-}^{l,B} \right|^{2}+\left( \left| t_{--}^{l,B} \right|^{2}-\left| t_{+-}^{l,B} \right|^{2} \right)\cos\delta-2\left| t_{--}^{l,B} \right|\left| t_{+-}^{l,B} \right|\sin[arg\left( t_{+-}^{l,B} \right)-arg\left( t_{--}^{l,B} \right)+2\alpha]\sin\delta\right\}$$

$=\frac{1}{2}\left\{ A_{2}^{l,B}+\sqrt{{(C_{3}^{l,B})}^{2}+{(C_{4}^{l,B})}^{2}}\cos(\delta+\phi_{--}^{l,B}) \right\}$ , (S10b)

$$T_{+-}^{l,B}=E_{+-}^{l,B}{\cdot E}_{+-}^{l,B*}$$

$$=\frac{1}{2}\left\{ \left| t_{--}^{l,B} \right|^{2}+\left| t_{+-}^{l,B} \right|^{2}-\left( \left| t_{--}^{l,B} \right|^{2}-\left| t_{+-}^{l,B} \right|^{2} \right)\cos\delta+2\left| t_{--}^{l,B} \right|\left| t_{+-}^{l,B} \right|\sin[arg\left( t_{+-}^{l,B} \right)-arg\left( t_{--}^{l,B} \right)+2\alpha]\sin\delta\right\}$$

$=\frac{1}{2}\left\{ A_{2}^{l,B}+\sqrt{{(C_{3}^{l,B})}^{2}+{(C_{4}^{l,B})}^{2}}\cos(\delta+\phi_{+-}^{l,B}) \right\}$ , (S10c)

$$T_{-+}^{l,B}=E_{-+}^{l,B}{\cdot E}_{-+}^{l,B*}$$

$$=\frac{1}{2}\left\{ \left| t_{++}^{l,B} \right|^{2}+\left| t_{-+}^{l,B} \right|^{2}-\left( \left| t_{++}^{l,B} \right|^{2}-\left| t_{-+}^{l,B} \right|^{2} \right)\cos\delta+2\left| t_{++}^{l,B} \right|\left| t_{-+}^{l,B} \right|\sin[arg\left( t_{-+}^{l,B} \right)-arg\left( t_{++}^{l,B} \right)-2\alpha]\sin\delta\right\}$$

$=\frac{1}{2}\left\{ A_{1}^{l,B}+\sqrt{{(C_{1}^{l,B})}^{2}+{(C_{2}^{l,B})}^{2}}\cos(\delta+\phi_{-+}^{l,B}) \right\}$ , (S10d)

where

$\left\{ \begin{aligned} A_{1}^{l,B}=\left| t_{++}^{l,B} \right|^{2}+\left| t_{-+}^{l,B} \right|^{2} \\ A_{2}^{l,B}=\left| t_{--}^{l,B} \right|^{2}+\left| t_{+-}^{l,B} \right|^{2} \\ C_{1}^{l,B}=\left| t_{++}^{l,B} \right|^{2}-\left| t_{-+}^{l,B} \right|^{2} \\ C_{2}^{l,B}=2\left| t_{++}^{l,B} \right|\left| t_{-+}^{l,B} \right|\sin[arg\left( t_{-+}^{l,B} \right)-arg\left( t_{++}^{l,B} \right)-2\alpha] \\ C_{3}^{l,B}=\left| t_{--}^{l,B} \right|^{2}-\left| t_{+-}^{l,B} \right|^{2} \\ C_{4}^{l,B}=2\left| t_{--}^{l,B} \right|\left| t_{+-}^{l,B} \right|\sin[arg\left( t_{+-}^{l,B} \right)-arg\left( t_{--}^{l,B} \right)+2\alpha] \end{aligned} \right.$ (S11)

and

$\phi_{++}^{l,B}=\phi_{-+}^{l,B}\pm\pi= {tan}^{-1} \frac{2\left| t_{++}^{l,B} \right|\left| t_{-+}^{l,B} \right|\sin[arg\left( t_{-+}^{l,B} \right)-arg\left( t_{++}^{l,B} \right)-2\alpha]}{\left| t_{++}^{l,B} \right|^{2}-\left| t_{-+}^{l,B} \right|^{2}}$ , (S12a)

$\phi_{--}^{l,B}=\phi_{+-}^{l,B}\pm\pi= {tan}^{-1} \frac{2\left| t_{--}^{l,B} \right|\left| t_{+-}^{l,B} \right|\sin[arg\left( t_{+-}^{l,B} \right)-arg\left( t_{--}^{l,B} \right)+2\alpha]}{\left| t_{--}^{l,B} \right|^{2}-\left| t_{+-}^{l,B} \right|^{2}}$ . (S12b)

It is clear from Eqs. (S7) and (S10) that the transmittance for the metasurface-sapphire system exhibits BI oscillations, similar to that of the sapphire slab but with a phase shift. However, as we are interested in the relative measurement to eliminate systematic errors and the numerical aperture (NA) effects of the optical system [5], the transmission phase for the metasurface-sapphire system is better characterized by the relative phase w.r.t. that of the sapphire slab, i.e. ${\Delta\phi}_{ij}^{l,F/B}$ = $\phi_{ij}^{l,F/B}-\phi_{ij}^{Sa}$. Thus, from Eqs. (S4), (S9), and (S12), the transmission phase for the metasurface-sapphire system can be written as:

${\Delta\phi}_{++}^{l,F}(\alpha)={\Delta\phi}_{+-}^{l,F}(\alpha)={tan}^{-1} \frac{2\left| t_{++}^{l,F} \right|\left| t_{+-}^{l,F} \right|\sin\left[ arg\left( t_{+-}^{l,F} \right)-arg\left( t_{++}^{l,F} \right)-2\alpha\right]}{\left| t_{++}^{l,F} \right|^{2}-\left| t_{+-}^{l,F} \right|^{2}}$ , (S13a)

${\Delta\phi}_{--}^{l,F}(\alpha)={\Delta\phi}_{-+}^{l,F}(\alpha)={tan}^{-1} \frac{2\left| t_{--}^{l,F} \right|\left| t_{-+}^{l,F} \right|\sin\left[ arg\left( t_{-+}^{l,F} \right)-arg\left( t_{--}^{l,F} \right)+2\alpha\right]}{\left| t_{--}^{l,F} \right|^{2}-\left| t_{-+}^{l,F} \right|^{2}}$, (S13b)

${\Delta\phi}_{++}^{l,B}(-\alpha)={\Delta\phi}_{-+}^{l,B}(-\alpha)={tan}^{-1} \frac{2\left| t_{++}^{l,B} \right|\left| t_{-+}^{l,B} \right|\sin[arg\left( t_{-+}^{l,B} \right)-arg\left( t_{++}^{l,B} \right)-2\alpha]}{\left| t_{++}^{l,B} \right|^{2}-\left| t_{-+}^{l,B} \right|^{2}}$ , (S13c)

${\Delta\phi}_{--}^{l,B}(-\alpha)={\Delta\phi}_{+-}^{l,B}(-\alpha)= {tan}^{-1} \frac{2\left| t_{--}^{l,B} \right|\left| t_{+-}^{l,B} \right|\sin[arg\left( t_{+-}^{l,B} \right)-arg\left( t_{--}^{l,B} \right)+2\alpha]}{\left| t_{--}^{l,B} \right|^{2}-\left| t_{+-}^{l,B} \right|^{2}}$ . (S13d)

Note that the relative transmission phase has a period of π such that the sign reveres when the base angle *α* changes by $\pm$ π/2, i.e.

${\Delta\phi}_{ij}^{l,F/B}\left( \alpha\right)= -{\Delta\phi}_{ij}^{l,F/B}\left( \alpha\pm\frac{\pi}{2} \right)$, (S14)

for $i,j=+/-.$ Moreover, according to the Lorentz Reciprocity Lemma theorem [1], $t_{ii}^{F}= t_{ii}^{B}$ for $i=+/-$and $t_{ij}^{F}= t_{ji}^{B}$ for $i\neq j =+/-$. Thus it can be shown from Eqs. (S7) and (S10) that the forward and backward transmittance for the co-polarization components are the same, i.e.

$T_{ii}^{l,F}(\alpha)=T_{ii}^{l,B}(-\alpha)$ (S15a)

for $=+/-$ , and for the cross-polarization components:

$T_{ij}^{l,F}(\alpha)=T_{ji}^{l,B}(-\alpha)$ (S15b)

for $i\neq j =+/-$. Moreover, from Eqs. (S13a-S13d) for the relative transmission phase, one can show that:

${\Delta\phi}_{ii}^{l,F}(\alpha)= {\Delta\phi}_{ii}^{l,B}(-\alpha)$ (S16a)

for the co-polarization components and

${\Delta\phi}_{ij}^{l,F}\left( \alpha\right)= {\Delta\phi}_{ji}^{l,B}\left( -\alpha\right)$ (S16b)

for the cross-polarization components.

Furthermore, as the “atoms” **N** and **и** are mirror images of each other and 2D chiral [6], the transmission coefficients of the planar chiral **N**- and **и**-type Au sawtooth metasurfaces satisfy $t_{ii}^{N}= t_{jj}^{N^{-1}}$and $t_{ij}^{N}= t_{ji}^{N^{-1}}$ for $i\neq j =+/-$. Note that the base angle *α/-α* for the planar **N**-/**и**-type sawtooth metasurface under reflection along the $\hat{y}$-axis will be -*α/α* for the **и**-/**N**-type sawtooth metasurface. Thus, the transmittance of the **N**- and **и**-type Au sawtooth metasurfaces for the forward and backward directions are related:

$T_{ii}^{N,F/B}(\alpha)=T_{jj}^{N^{-1},F/B}(-\alpha)$, (S17a)

for $i=+/-$ and

$T_{ij}^{N,F/B}(\alpha)=T_{ji}^{N^{-1},F/B}(-\alpha)$, (S17a)

for $i\neq j =+/-$.

Furthermore, by replacing *α* by -*α* for the **и**-type Au sawtooth metasurface the relative transmission phase for the forward direction can be written as:

${\Delta\phi}_{++}^{N,F}(\alpha)={\Delta\phi}_{+-}^{N,F}(\alpha)= {tan}^{-1} \frac{2\left| t_{++}^{N,F} \right|\left| t_{+-}^{N,F} \right|\sin\left[ arg\left( t_{+-}^{N,F} \right)-arg\left( t_{++}^{N,F} \right)-2\alpha\right]}{\left| t_{++}^{N,F} \right|^{2}-\left| t_{+-}^{N,F} \right|^{2}}$, (S18a)

${\Delta\phi}_{--}^{N,F}(\alpha)={\Delta\phi}_{-+}^{N,F}(\alpha)= {tan}^{-1} \frac{2\left| t_{--}^{N,F} \right|\left| t_{-+}^{N,F} \right|\sin\left[ arg\left( t_{-+}^{N,F} \right)-arg\left( t_{--}^{N,F} \right)+2\alpha\right]}{\left| t_{--}^{N,F} \right|^{2}-\left| t_{-+}^{N,F} \right|^{2}}$ , (S18b)

${\Delta\phi}_{++}^{N^{-1},F}(-\alpha)={\Delta\phi}_{+-}^{N^{-1},F}(-\alpha)= {tan}^{-1} \frac{2\left| t_{++}^{N^{-1},F} \right|\left| t_{+-}^{N^{-1},F} \right|\sin\left[ arg\left( t_{+-}^{N^{-1},F} \right)-arg\left( t_{++}^{N^{-1},F} \right)+2\alpha\right]}{\left| t_{++}^{N^{-1},F} \right|^{2}-\left| t_{+-}^{N^{-1},F} \right|^{2}}$

$= {tan}^{-1} \frac{2\left| t_{--}^{N,F} \right|\left| t_{-+}^{N,F} \right|\sin\left[ arg\left( t_{-+}^{N,F} \right)-arg\left( t_{--}^{N,F} \right)+2\alpha\right]}{\left| t_{--}^{N,F} \right|^{2}-\left| t_{-+}^{N,F} \right|^{2}}$, (S18c)

${\Delta\phi}_{--}^{N^{-1},F}(-\alpha)={\Delta\phi}_{-+}^{N^{-1},F}(-\alpha)= {tan}^{-1} \frac{2\left| t_{--}^{N^{-1},F} \right|\left| t_{-+}^{N^{-1},F} \right|\sin\left[ arg\left( t_{-+}^{N^{-1},F} \right)-arg\left( t_{--}^{N^{-1},F} \right)-2\alpha\right]}{\left| t_{--}^{N^{-1},F} \right|^{2}-\left| t_{-+}^{N^{-1},F} \right|^{2}}$

$= {tan}^{-1} \frac{2\left| t_{++}^{N,F} \right|\left| t_{+-}^{N,F} \right|\sin\left[ arg\left( t_{+-}^{N,F} \right)-arg\left( t_{++}^{N,F} \right)-2\alpha\right]}{\left| t_{++}^{N,F} \right|^{2}-\left| t_{+-}^{N,F} \right|^{2}}$ , (S18d)

leading to:

${\Delta\phi}_{++}^{N,F}(\alpha)={\Delta\phi}_{+-}^{N,F}(\alpha)={\Delta\phi}_{--}^{N^{-1},F}(-\alpha)={\Delta\phi}_{-+}^{N^{-1},F}(-\alpha)$ (S19a)

and

${\Delta\phi}_{--}^{N,F}(\alpha)={\Delta\phi}_{-+}^{N,F}(\alpha)={\Delta\phi}_{++}^{N^{-1},F}(-\alpha)={\Delta\phi}_{+-}^{N^{-1},F}(-\alpha)$. (S19b)

Similarly, the relative transmission phase for **N**- and **и**-type chiral metasurface-sapphire system for the backward direction can be written as:

${\Delta\phi}_{++}^{N,B}(-\alpha)={\Delta\phi}_{-+}^{N,B}(-\alpha)={\Delta\phi}_{--}^{N^{-1},B}(\alpha)={\Delta\phi}_{+-}^{N^{-1},B} (\alpha)$ (S20a)

and

$\Delta\phi_{--}^{N,B}(-\alpha)={\Delta\phi}_{+-}^{N,B}(-\alpha)={\Delta\phi}_{++}^{N^{-1},B}(\alpha)={\Delta\phi}_{-+}^{N^{-1},B} (\alpha)$. (S20b)

Following the (traditional) asymmetric transmission notation for chiral metasurface, ${\Delta T}_{ij}^{l}(\alpha)$= $T_{ij}^{l,F}(\alpha)-T_{ij}^{l,B}(-\alpha)$ [7], we define the asymmetric transmission phase for our **N**- and **и**-type Au sawtooth metasurface-sapphire system ${\Delta\boldsymbol{\Phi}}_{ij}^{l}$ as:

${\Delta\boldsymbol{\Phi}}_{ij}^{l}$ $(\alpha)$= ${\Delta\phi}_{ij}^{l,F}(\alpha)-{\Delta\phi}_{ij}^{l,B}(-\alpha)$ . (S21)

Then, it can be shown from Eq. (S16) that:

${\Delta\boldsymbol{\Phi}}_{ii}^{l} {\left( \alpha\right)= \Delta\phi}_{ii}^{l,F}(\alpha)- {\Delta\phi}_{ii}^{l,B}(-\alpha)=0$ (S22a)

for $i=+/-$ and

${\Delta\boldsymbol{\Phi}}_{ij}^{l}(\alpha) {= \Delta\phi}_{ij}^{l,F}(\alpha)- {\Delta\phi}_{ij}^{l,B}(-\alpha) {= -(\Delta\phi}_{ji}^{l,F}(\alpha)- {\Delta\phi}_{ji}^{l,B}(-\alpha))= -{\Delta\boldsymbol{\Phi}}_{ji}^{l} (\alpha)$ (S22b)

for $i\neq j =+/-$. Thus the asymmetric transmission phase for the **N**-/**и**-type Au sawtooth metasurface-sapphire system is zero for the co-polarization components but non-zero and antisymmetric for the cross-polarization components. Furthermore, it can be shown from Eqs. (S19) and (S20) that:

${\Delta\boldsymbol{\Phi}}_{ij}^{N} {\left( \alpha\right)= \Delta\phi}_{ij}^{N,F}(\alpha)- {\Delta\phi}_{ij}^{N,B}(-\alpha) {= (\Delta\phi}_{ji}^{N^{-1},F}(-\alpha)- {\Delta\phi}_{ji}^{N^{-1},B}(\alpha))= {\Delta\boldsymbol{\Phi}}_{ji}^{N^{-1}}(-\alpha)= -{\Delta\boldsymbol{\Phi}}_{ij}^{N^{-1}}(-\alpha)$ (S23)

for $i,j =+/-$. Eqs. (S22) and (S23) summarize well the rich asymmetry transmission phase properties of the **N**-/**и**-type Au sawtooth metasurface-sapphire system.

1. **Experimental transmittance and transmission phase**

In general, the experimental transmittance of the metasurface-sapphire system can be represented by [8]:

$T_{ij}^{l}=A_{ij}+ B_{ij}\cos\left( \frac{2\pi}{\lambda_{ij}^{l}}\left( n_{o}-n_{e} \right)L+\phi_{ij}^{l} \right)$, (S24)

where $A_{ij}$ and $B_{ij}$ are slowly varying functions of the wavenumber due to the dispersion of the Au sawtooth metasurface and the sapphire slab. Eq. (S24) exhibits BI oscillations with transmission peaks/troughs for constructive/destructive interference satisfying the condition:

$\frac{2\pi}{\lambda_{ij}^{l,m}}\left( n_{o}-n_{e} \right)L+\phi_{ij}^{l,m}=2m\pi$, (S25)

with $\lambda_{ij}^{l,m}$ the wavelength of the $m^{th}$/${(m+\frac{1}{2})}^{th}$ interference order of the metasurface-sapphire system and *l* = Sa/*N*/*N^-1^* denotes the sapphire/**N**-/**и**-type slab/chiral metasurface. Eq. (S25) can be rewritten with the phase normalized by 2π as:

$m=\frac{1}{\lambda_{ij}^{l,m}}\left( n_{o}-n_{e} \right)L+\phi_{ij}^{l,m}$. (S26)

(For simplicity, the 2π is dropped for the normalized phase.) To eliminate systematic errors of the optical system we express the transmission phase of the metasurface-sapphire system w.r.t. that of the sapphire slab. Considering the same BI interference order for the metasurface-sapphire system and the sapphire slab, we have:

$\left[ \frac{1}{\lambda_{ij}^{l,m}}\left( n_{o}-n_{e} \right)L+\phi_{ij}^{l,m} \right]=\left[ \frac{1}{\lambda_{ij}^{sa,m}}\left( n_{o}-n_{e} \right)L+\phi_{ij}^{Sa,m} \right]$ (S27)

Furthermore, Eq. (S27) can be rewritten as:

${\Delta\phi}_{ij}^{l,m}=\phi_{ij}^{l,m}- \phi_{ij}^{Sa,m}=\left[ \frac{1}{\lambda_{ij}^{sa,m}}-\frac{1}{\lambda_{ij}^{l,m}} \right]\left( n_{o}-n_{e} \right)L$ . (S28)

However, from Eq. (S24) for the sapphire slab, we have $\left( n_{o}-n_{e} \right)L=\frac{1}{\frac{1}{\lambda_{ij}^{sa,m+1}} - \frac{1}{\lambda_{ij}^{sa,m}}}$ for $\phi_{ij}^{Sa,m+1}=\phi_{ij}^{Sa,m}$. Thus the experimental transmission phase of the metasurface-sapphire system can be written as:

${\boldsymbol{\Delta}\boldsymbol{\phi}}_{\boldsymbol{ij}}^{\boldsymbol{l}\boldsymbol{,m}}\boldsymbol{=}\left( \frac{\frac{\boldsymbol{1}}{\boldsymbol{\lambda}_{\boldsymbol{ij}}^{\boldsymbol{sa,m}}}\boldsymbol{-}\frac{\boldsymbol{1}}{\boldsymbol{\lambda}_{\boldsymbol{ij}}^{\boldsymbol{l,m}}}}{\frac{\boldsymbol{1}}{\boldsymbol{\lambda}_{\boldsymbol{ij}}^{\boldsymbol{sa,m+1}}}\boldsymbol{-}\frac{\boldsymbol{1}}{\boldsymbol{\lambda}_{\boldsymbol{ij}}^{\boldsymbol{sa,m}}}} \right)$ . (S29)

Eq. (S29) is called the peak shift method.

1. **Transmittance and asymmetric transmission of Au sawtooth metasurface-sapphire system**

The **N**-type Au sawtooth chiral metasurface was fabricated on a “non-birefringent” substrate (0.43 mm c-cut sapphire plate) using an e-beam evaporation technique for the Au film (30 nm thick) deposition and then an ion-beam direct-write technique to generate the Au sawtooth nanoarrays. (Note that the c-cut sapphire plate, though a birefringent material, has an optical axis perpendicular to the plane of the plate so that there is no birefringent effect for normal incidence.) The Au sawtooth metasurface was then placed on the top of an *L* = 2 mm a-cut sapphire birefringent crystal plate to form the Au sawtooth metasurface-sapphire system. The transmittance of the **N**-type Au sawtooth metasurface-sapphire system exhibits BI oscillations as shown in Figure S1 for both the forward (left column) and backward incidence (middle column) with transmission peak/trough positions depending on the base angle of the sawtooth nanoarray *α*. Nevertheless, the transmittance shows, within the experimental errors, $T_{++}^{l,F}$ ($T_{--}^{l,F}$) is $\pi$ out of phase with $T_{+-}^{l,F}$ ($T_{-+}^{l,F}$), and $T_{++}^{l,B}$ ($T_{--}^{l,B}$) is $\pi$ out of phase with $T_{-+}^{l,B}$ ($T_{+-}^{l,B}$) as shown in the insets, in very agreement with Eqs. (S7) and (S10). However, the amplitude for the forward transmittance is different from that of the backward transmittance due mostly to the effect of the substrate (including the asymmetric losses) which is breaking the symmetry of the Au sawtooth metasurface. This will generate undesirable results as shown in the third column of Figure S1 for the asymmetric transmission defined simply by the difference of the forward and backward transmittance (${\Delta T}_{ij}^{l}(\alpha)= T_{ij}^{l,F}(\alpha)-T_{ij}^{l,B}(-\alpha)$) for various base angles. For example, it is clear that ${\Delta T}_{++}^{N}(\alpha)= T_{++}^{N,F}(\alpha)-T_{++}^{N,B}(-\alpha)$ (blue solid curve) is non-zero, similarly for ${\Delta T}_{--}^{N}(\alpha)= T_{--}^{N,F}(\alpha)-T_{--}^{N,B}(-\alpha)$ (red dashed curve), in disagreement with Eq. (S15a). The effect of the substrate can be reduced/removed by placing a matching substrate on the top of the Au sawtooth metasurface as reported earlier to make the sample reciprocal [9]. Another simpler method to rectify the substrate effect is to re-normalize the backward transmittance to that of the forward transmittance before the subtraction by:

$T_{norm}^{l,B}=(T^{l,B}-T_{bot\_env}^{l,B}$) ${(T}_{top\_env}^{l,F}-T_{bot\_env}^{l,F}$) /${(T}_{top\_env}^{l,B}-T_{bot_{env}}^{l,B})$ + $T_{bot\_env}^{l,F}$ , (S30)

where $T_{top\_env}^{l,F/B}$ and $T_{bot\_env}^{l,F/B}$ are the top and bottom envelopes of the transmittance, respectively. The last column of Figure S1 shows the asymmetric transmission after the re-normalization. Now the substrate effect is much reduced, though the co-polarization asymmetric transmission is still non-zero due mainly to errors in finding the envelope functions. Despite the noise, the asymmetric transmission for the cross-polarization components are now more prominent, e.g. as indicated by the green envelope in the graph for $\alpha={120}^{^{\circ}}$ for the ${\Delta T}_{+-}^{N}$ asymmetric transmission. Moreover, it shows a maximum asymmetric transmission amplitude ~0.4 at wavenumber 1/*λ* ~ 1.36 μm^-1^ as indicated by the black arrow.


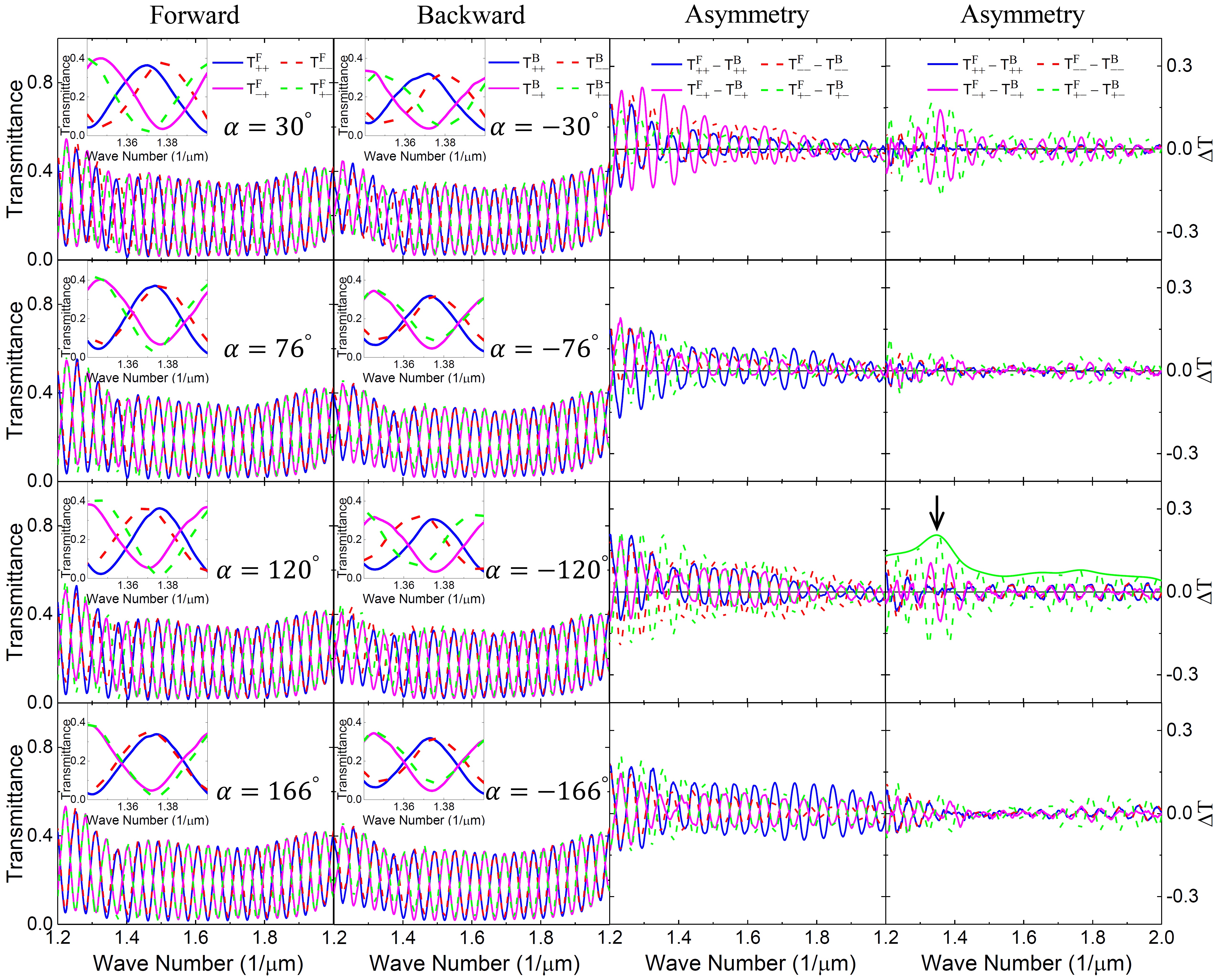


**Figure S1:** Experimental transmittance for the **N**-type Au sawtooth metasurface-sapphire with various base angles $\alpha$. The first column is for the forward incidence, the second column is for the backward incidence, the third column is the uncorrected asymmetric transmission, and the last column is the corrected asymmetric transmission. The insets are the transmittance on an expanded scale for about one birefringence interference period. The solid green curve is the envelope for the ${\Delta T}_{+-}^{N}$. The black arrow shows the extremum of the asymmetric transmission.

To cross-check our experimental results, we perform corresponding full-wave simulations for the **N**-type Au sawtooth metasurface-sapphire system as shown in Figure S1. To simplify the simulations, the metasurface substrate is not included in the metasurface-sapphire system. Now, the asymmetric transmission, last column in Figure S2, shows clearly within the numerical errors, that ${\Delta T}_{++}^{N}={\Delta T}_{--}^{N}=0$ for the co-polarization components (blue and red solid curves), in good agreement with Eq. (S15a). Moreover, the asymmetric transmission for the cross-polarization components (magenta solid and green dashed curves in Figure S2) exhibit a maximum as indicated by the black arrow for the ${\Delta T}_{+-}^{N}$ envelope (green solid curve) in the graph for $\alpha={120}^{^{\circ}}$ of the last column in Figure S2. Note that the peak asymmetric transmission (amplitude ~0.3 at wavenumber 1/*λ* ~ 1.42 μm^-1^) is shifted slightly from that of the experiment due to the substrate effect and also the physical parameters (dimensions of the Au sawtooth nanoarrays and dielectric constants of the materials) used in the simulations may not match those of the experiment perfectly. Despite the difference, the simulations confirm the experimental results very well.


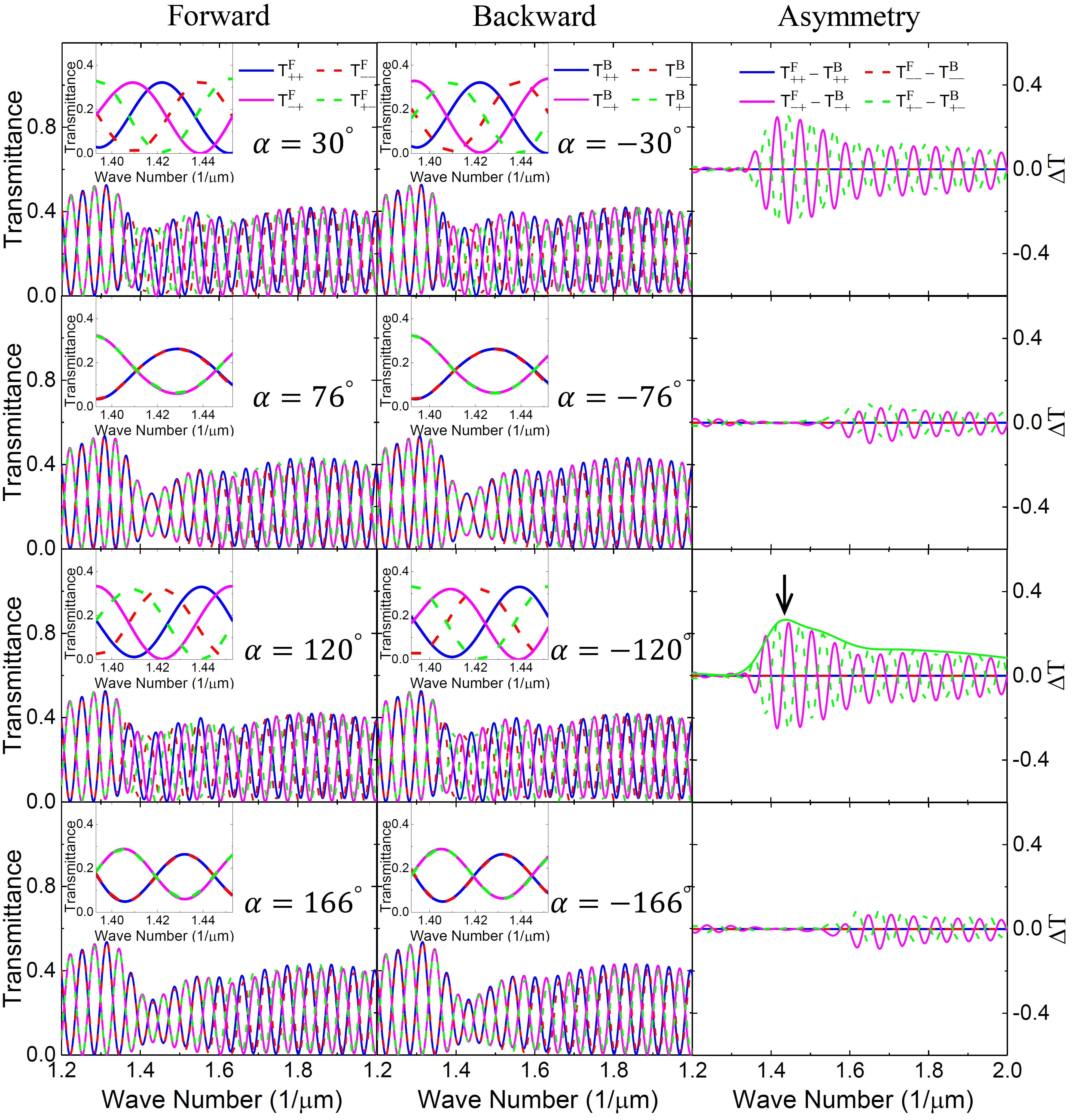


**Figure S2:** Simulation results for the **N**-type Au sawtooth metasurface-sapphire system for forward (left column), backward (middle column), and asymmetric transmission (right column). The insets are the transmittance on an expanded scale for about one birefringence interference period. The solid green curve is the envelope for the ${\Delta T}_{+-}^{N}$. The black arrow shows the extremum of the asymmetric transmission.

The asymmetric transmission for the **и**-type Au sawtooth metasurface-sapphire system exhibits similar results as those of the **N**-type Au sawtooth metasurface-sapphire system as shown in Figures S3 and S4.


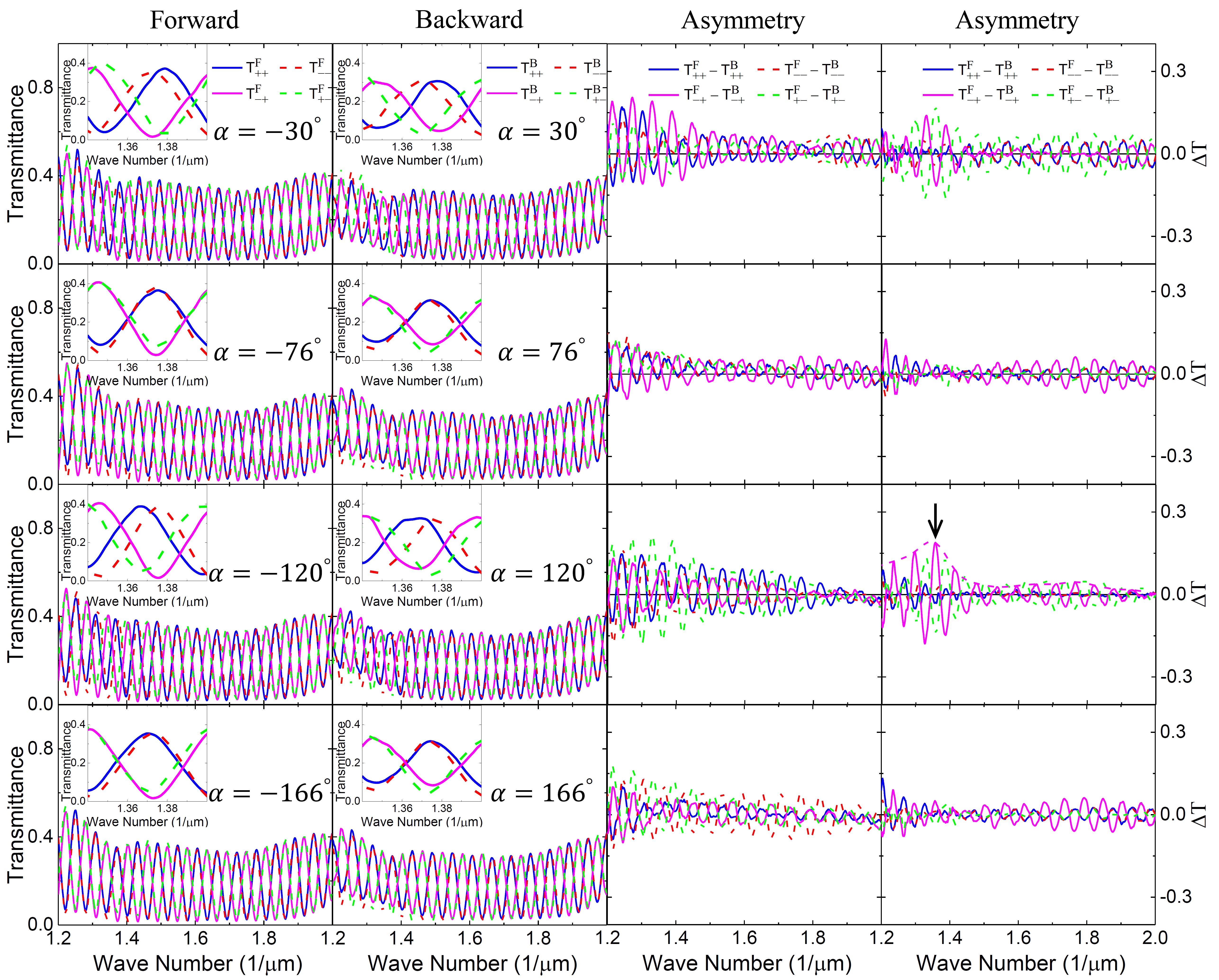


**Figure S3:** Experimental transmittance for the $\text{и}$-type Au sawtooth metasurface-sapphire system with various base angles $\alpha$. The first column is for the forward incidence, the second column is for the backward incidence, the third column is the uncorrected asymmetric transmission, and the last column is the corrected asymmetric transmission. The insets are the transmittance on an expanded scale for about one birefringence interference period. The dashed magenta curve is the envelope for the ${\Delta T}_{-+}^{N^{-1}}$. The black arrow shows the extremum of the asymmetric transmission.


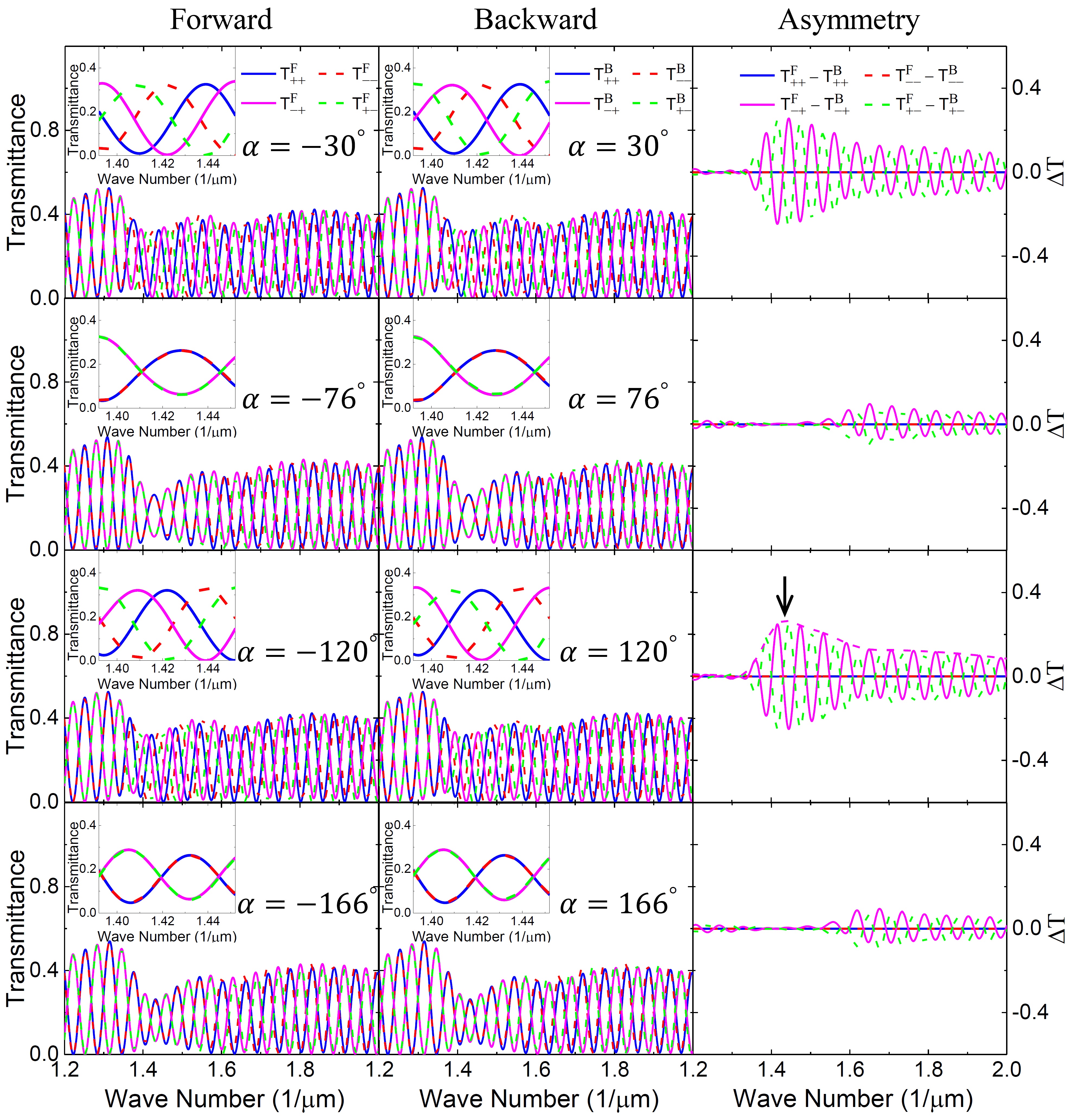


**Figure S4:** Simulation results for the $\text{и}$-type Au sawtooth metasurface-sapphire system for forward (left column), backward (middle column), and asymmetric transmission (right column). The insets are the transmittance on an expanded scale for about one birefringence interference period. The dashed magenta curve is the envelope for the ${\Delta T}_{-+}^{N^{-1}}$ . The black arrow shows the extremum of the asymmetric transmission.

1. **Resonances of Au sawtooth metasurface**

Figure S5 shows the transmittance of the Au sawtooth metasurface. The transmittance for the co-polarization components is much larger than that of the cross-polarization components such that the resonances, at wavenumbers 1/*λ* ~1.37 μm^-1^ and 1/*λ* ~1.75 μm^-1^, for the Au sawtooth metasurface are visible as indicated by the black arrows. However, the amplitudes of the forward and backward transmittance for the co-polarization components are slightly different due to the effects of the substrate of the Au sawtooth metasurface. It is possible to reduce/remove the substrate effect by adding a matching substrate on the Au sawtooth metasurface as reported earlier [9]. Nevertheless, the cross-polarization components exhibit chiral responses, e.g. ${\Delta T}_{+-}^{N}$~ -0.04 and ${\Delta T}_{-+}^{N}$ ~ 0.02 at 1/*λ* ~1.37 μm^-1^, of the Au sawtooth metasurface, as shown in the last column of Figure S5. As the sapphire slab is absent, there is no tunning parameter for the asymmetric transmission for the Au sawtooth metasurface alone.

**
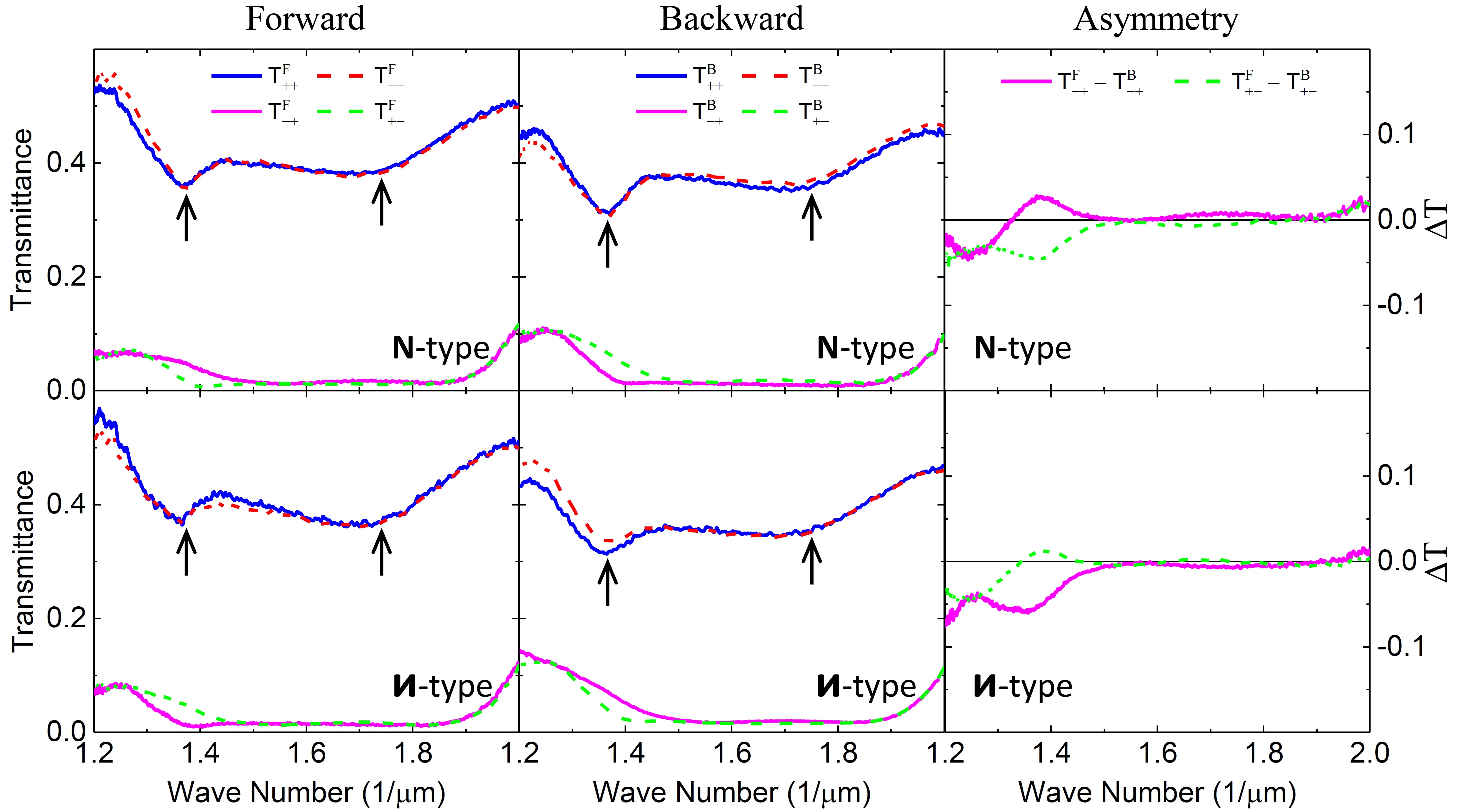
**

**Figure S5:** Transmittance and asymmetric transmission for **N**-,and $\text{и}$-type Au sawtooth metasurfaces$.$ The black arrows are resonances of the Au sawtooth metasurfaces.

1. **Asymmetric transmission phase of Au sawtooth metasurface-sapphire system**

The first and second columns of Figure S6 show the experimental transmission phase relative to those of the sapphire slab for the **N**-type Au sawtooth metasurface-sapphire system for various base angles *α*. It is noted from the graphs in Figure S6 that the relative transmission phases for the **N**-type Au sawtooth metasurface are the same, independent of the base angle, for the same transmitted component for the forward incidence and also for the same incident component for the backward incidence, i.e. ${\Delta\phi}_{ii}^{N,F}={\Delta\phi}_{ij}^{N,F}$ and ${\Delta\phi}_{ii}^{N,B}={\Delta\phi}_{ji}^{N,B}$ for $i\neq j =+/-$, in good agreement with Eq. (S13). The last column of Figure S6 is the asymmetric transmission phase defined in Eq. (S21) as ${\Delta\boldsymbol{\Phi}}_{ij}^{N}(\alpha) = {\Delta\phi}_{ij}^{N,F}(\alpha)-{\Delta\phi}_{ij}^{N,B}(-\alpha)$ for the **N**-type Au sawtooth metasurface-sapphire system. The asymmetric transmission phase is almost zero within the experimental errors for the co-polarization components (blue and red dots in Figure S6), in good agreement with Eq. (S22a). Importantly, the asymmetric transmission phase is nearly antisymmetric for the cross-polarization components (green and magenta dots in Figure S6), also in good agreement with Eq. (S22b). Moreover, the asymmetric transmission phase for the cross-polarization components exhibit local maxima (indicated by the black arrows in the graphs for $\alpha={120}^{^{\circ}},$ one at wavenumber 1/*λ* ~ ${1.37\mu m}^{-1}$ and the other at 1/*λ* ~ ${1.75\mu m}^{-1})$, corresponding to the resonances discussed in Section 4 above. Importantly, the asymmetric transmission phase can be tuned by simply controlling the base angle $\alpha$ of sawtooth nanoarray of the metasurface-sapphire system.

**
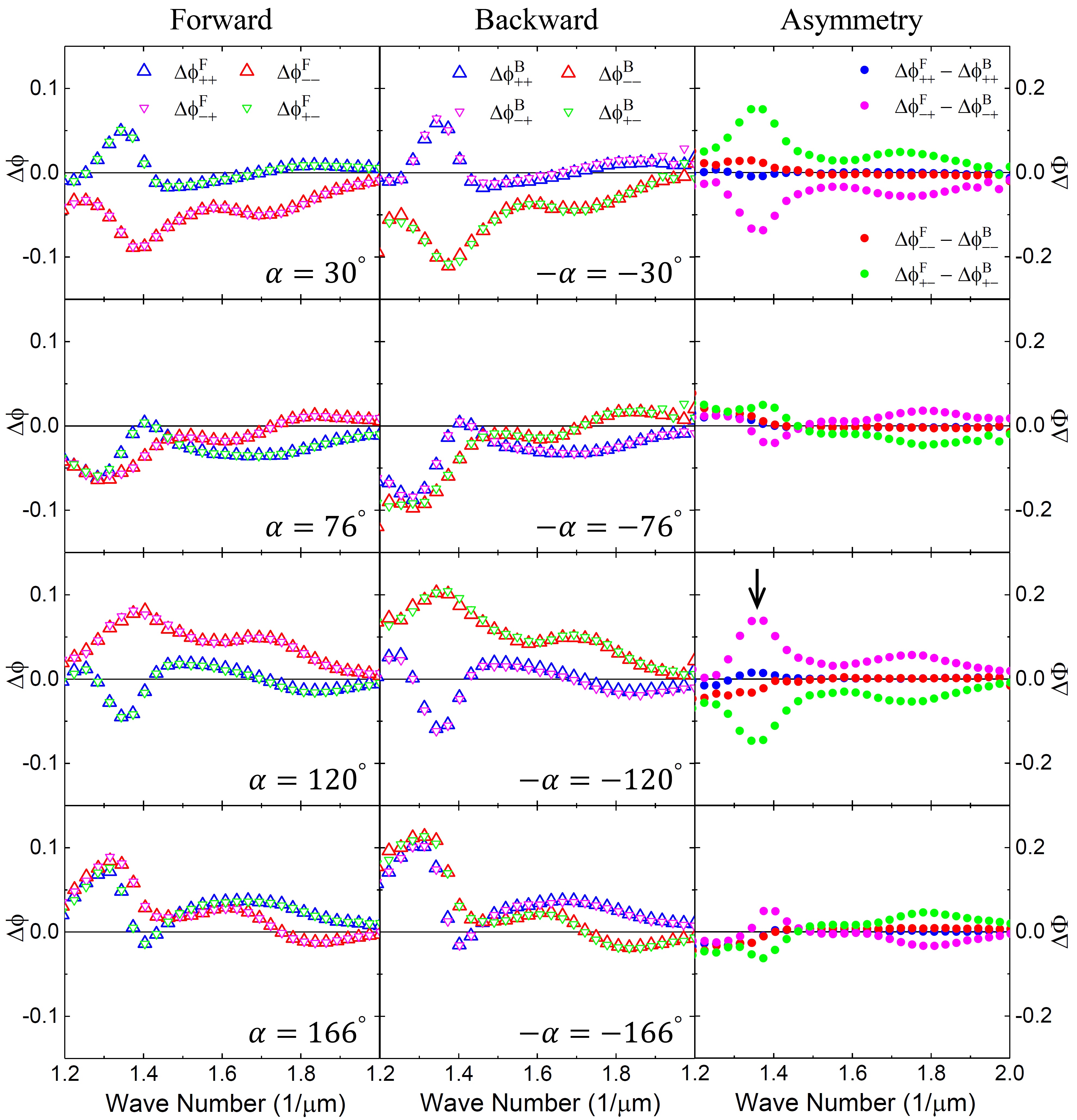
**

**Figure S6:** Experimental asymmetric transmission phase for the **N**-type Au sawtooth metasurface-sapphire system with various base angles $\alpha$. The left column is for the relative forward transmission phase, the middle column is for the relative backward transmission phase, and the right column is the asymmetric transmission phase. The black arrow shows the extremum of the asymmetric transmission phase at 1/*λ* ~ ${1.37\mu m}^{-1}$.

Figure S7 shows the corresponding simulation results for the **N**-type Au sawtooth metasurface-sapphire system in Figure S6. The simulated asymmetric transmission phases are exactly zero within the numerical errors for the co-polarization components (blue and red dots in Figure S7), in perfect agreement with Eq. (S22a). As for the cross-polarization components, the simulations exhibit very similar results as the experimental results except there is a shift of the local maxima (one at wavenumber 1/*λ* ~ ${1.42\mu m}^{-1}$ and the other at 1/*λ* ~ ${1.67\mu m}^{-1})$ as indicated by the black arrow in Figure S7 for the local maximum at 1/*λ* ~ ${1.42\mu m}^{-1}$ . The simulations support strongly that one can tune the asymmetric transmission phase of the metasurface-sapphire system simply by controlling the base angle *α*.


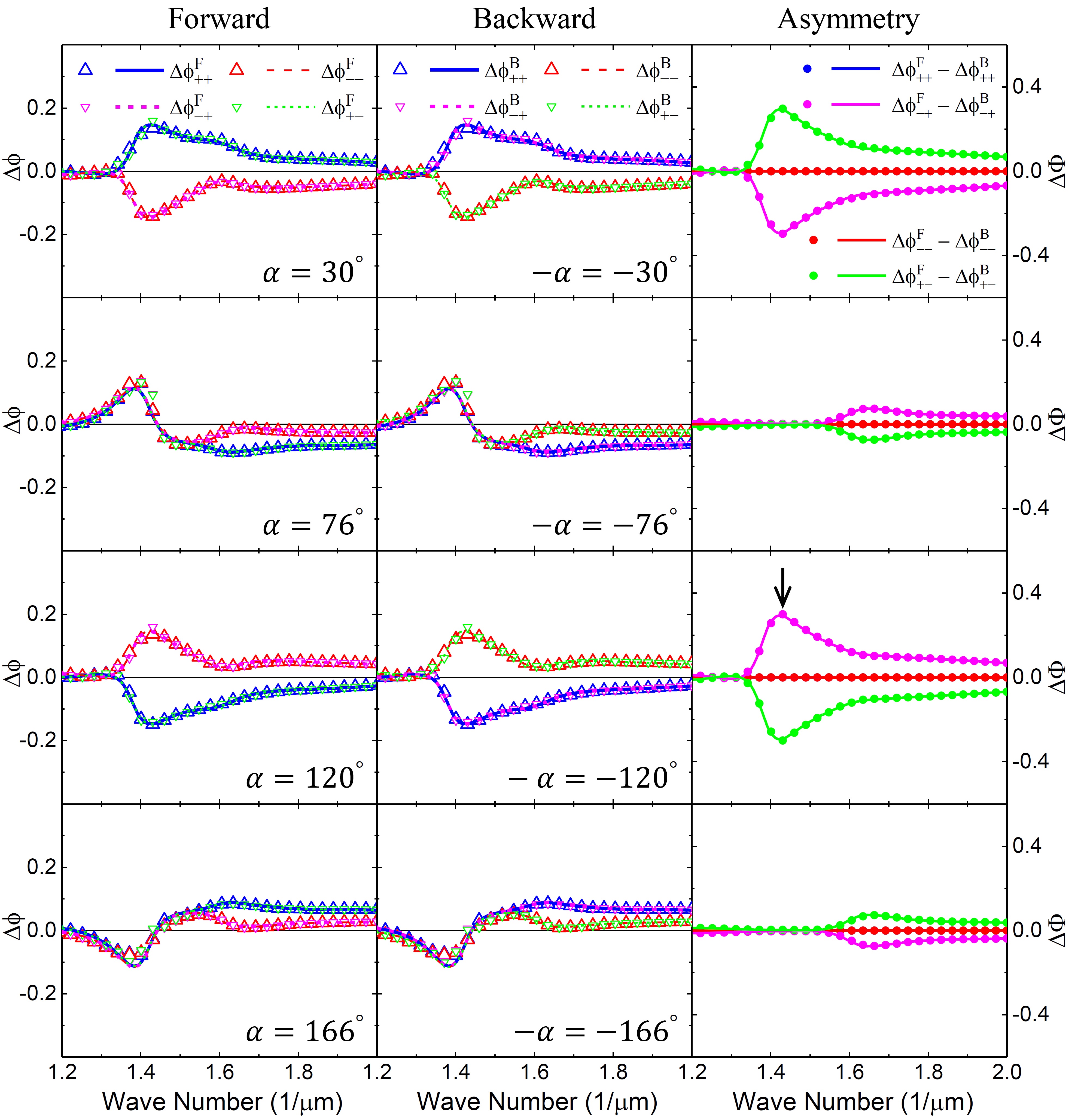


**Figure S7:** Simulation results for the **N**-type Au sawtooth metasurface-sapphire system for forward (left column), backward (middle column), and asymmetric transmission phase (right column). Dots are obtained from the peak shift method using Eqs. (S21) and (S29) and curves are from direct calculations using Eqs. (S13) and (S21). The black arrow shows the extremum of the asymmetric transmission phase at 1/*λ* ~ ${1.42\mu m}^{-1}$.

Figures S8 and S9 show results for the **и**-type Au sawtooth metasurface-sapphire system, complementing well those for the **N**-type Au sawtooth metasurface-sapphire system, as the **и**- and **и**-type Au sawtooth arrays are complementary.

**
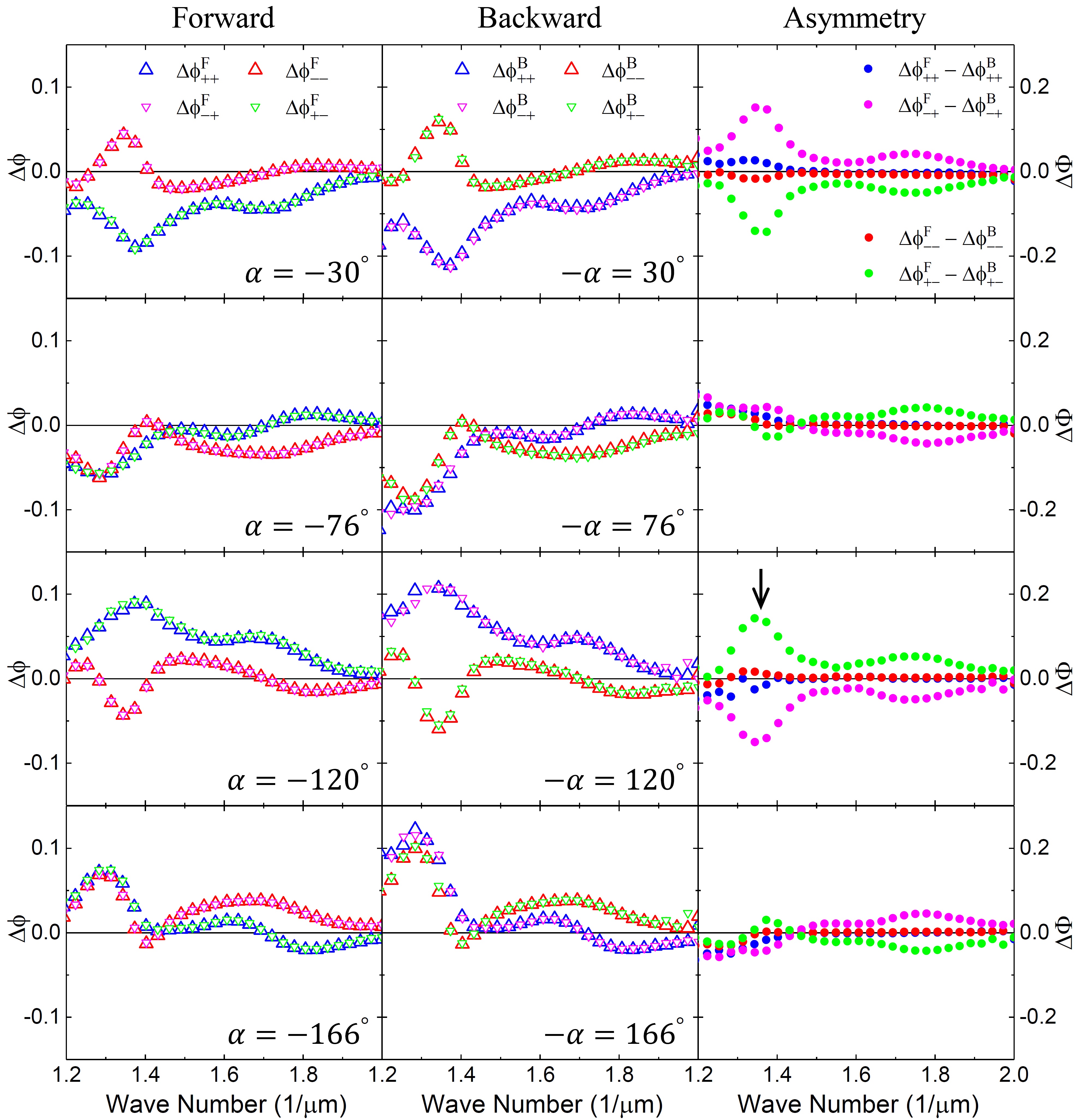
**

**Figure S8:** Experimental asymmetric transmission phase for the $\text{и}$-type Au sawtooth metasurface-sapphire system with various base angles $\alpha$. The left column is for the relative forward transmission phase, the middle column is for the relative backward transmission phase, and the right column is the asymmetric transmission phase. The black arrow shows the extremum of the asymmetric transmission phase at 1/*λ* ~ ${1.37\mu m}^{-1}$.


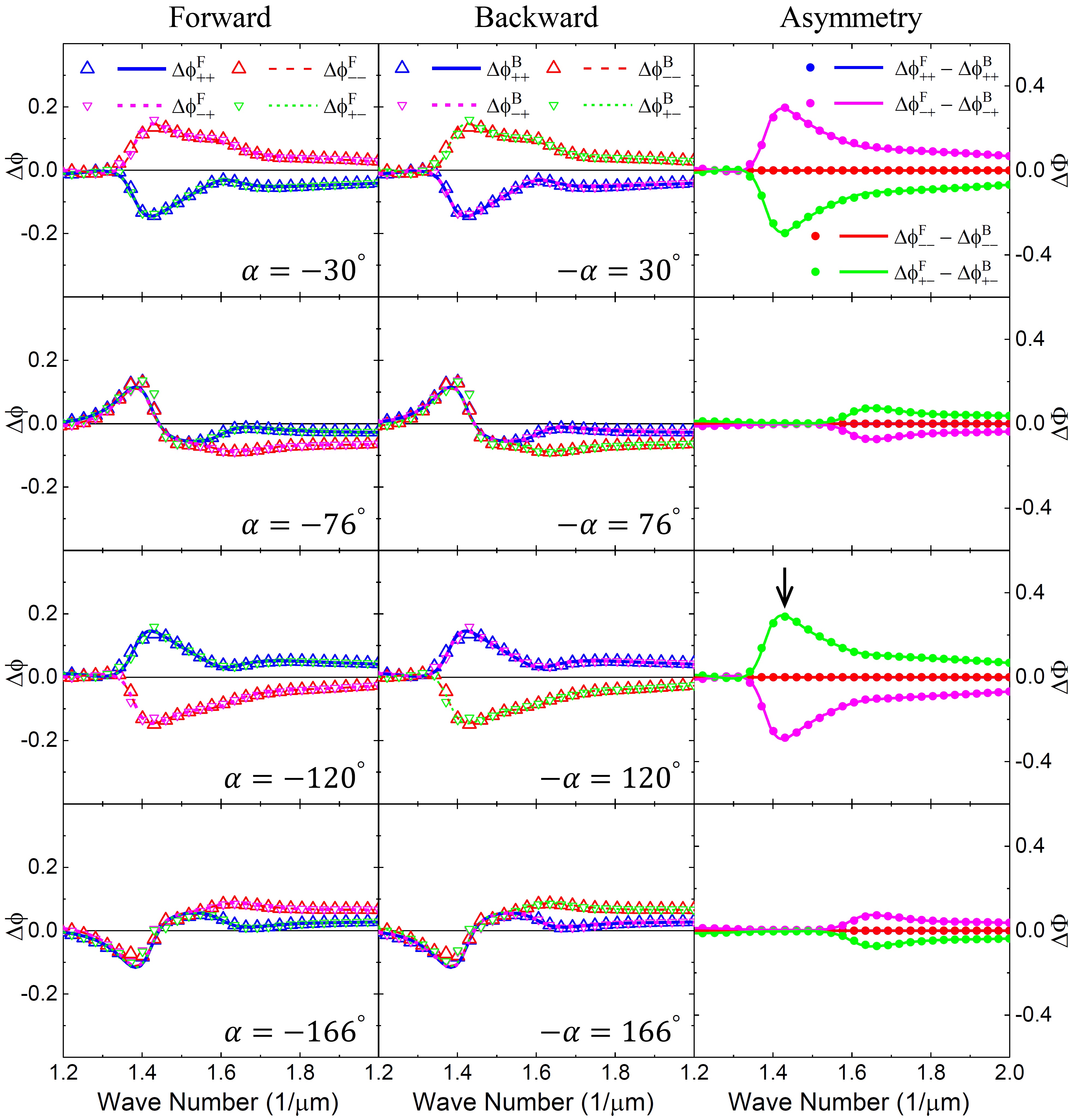


**Figure S9:** Simulation results for the $\text{и}$-type Au sawtooth metasurface-sapphire system for forward (left column), backward (middle column), and asymmetric transmission phase (right column). Dots are obtained from the peak shift method using Eqs. (S21) and (S29) and curves are from direct calculations using Eqs. (S13) and (S21). The black arrows show the extremum of the asymmetric transmission phase at 1/*λ* ~ ${1.42\mu m}^{-1}$.

Figure S10 shows the experimental and simulation results of the asymmetric transmission phase for **N**- and $\text{и}$-type Au sawtooth metasurface-sapphire systems for various base angles $\alpha$ chosen for extreme responses for the resonance at wavenumber 1/*λ* ~1.75 μm^-1^, similar to Figure 3 in the main text for wavenumber 1/*λ* ~1.37 μm^-1^. Note that now maximum responses occur at $\alpha$ ~14^o^ and ~104^o^ as indicated by the black arrows. The simulations agree well with the experimental results except that the maximum asymmetric phase is not as obvious as the resonance at 1/*λ* ~1.37μm^-1^ and also shifted to wavenumber 1/*λ* ~1.67 μm^-1^ due to the effect of the Au sawtooth substrate and parameters used in the simulations may not match those of the experiment perfectly.

**
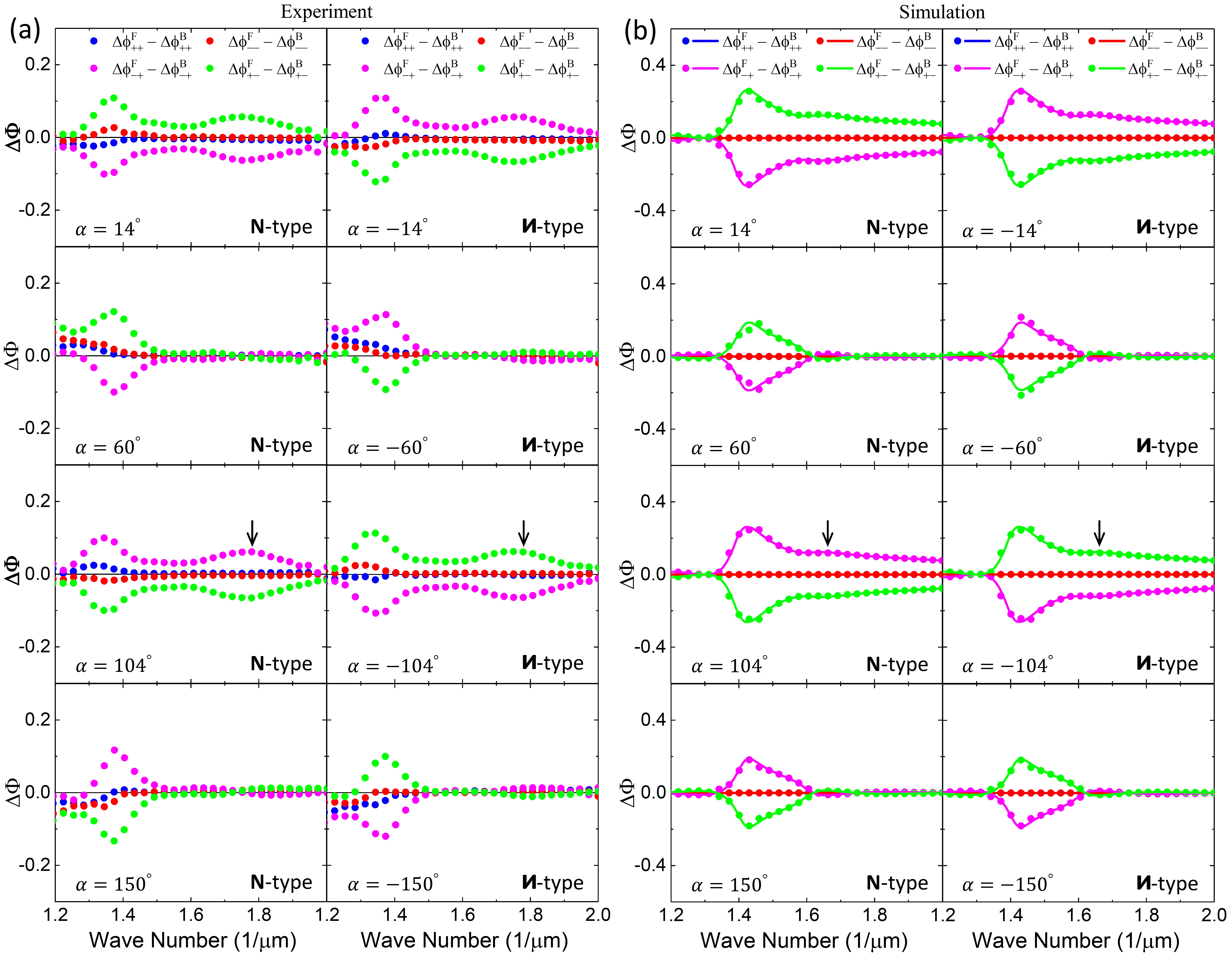
**

**Figure S10:** Asymmetric transmission phase of the **N**- and $\text{и}$-type Au sawtooth metasurfaces. (a) Experimental results of asymmetric transmission phase for **N**- and $\text{и}$-type Au sawtooth metasurface-sapphire systems for various control base angles $\alpha$chosen for extreme responses at wavenumber 1/*λ* ~1.75 μm^-1^. Black arrows indicate the local maximum. (b) Corresponding asymmetric transmission phase from the simulations. Solid dots are obtained from the peak shift method using Eqs. (S21) and (S29) and curves are from calculations using Eqs. (S13) and (S21). Black arrows indicate the local maximum at 1/*λ* ~1.67 μm^-1^.

1. **Asymmetric transmission of Au sawtooth metasurface-sapphire system**

Chiral materials are usually characterized by the asymmetric transmission defined as the difference between the forward and backward transmittance of circularly polarized light as described above in Sections 1 and 4. It is shown in Figure S5 that the asymmetric transmission for the Au sawtooth metasurface alone is relatively small and, importantly, cannot be altered once the As sawtooth nanoarrays are fabricated. However, our approach for the asymmetric transmission phase described in Sections 1 and 5 can be applied simultaneously to the study of asymmetric transmission. Figures S1 and S3 show the experimental results of the asymmetric transmission for the Au sawtooth metasurface-sapphire system at various base angles after rescaling the backward transmittance to that of the forward transmittance. Figure S11(a) shows the asymmetric transmission for the **N**- and $\text{и}$-type Au sawtooth metasurface-sapphire system for all base angles at the resonance wavenumber 1/*λ* ~1.36 μm^-1^ and 1/*λ* ~1.74 μm^-1^. The asymmetric transmission shows oscillatory behavior with amplitudes similar to those for the asymmetric transmission phase as shown in Figure 4(a), even though they are not as symmetric as those for the asymmetric transmission phase. Displayed also in Figure S11(a) are the asymmetric transmission (magenta and green dashed lines) for the Au sawtooth metasurface without the sapphire slab. It is clear that the asymmetric transmission for the metasurface-sapphire system is much larger and, more importantly, can be tuned by controlling the base angle *α*. The sapphire slab exhibits some kind of spatially dependent amplifications for the asymmetric transmission of the Au sawtooth metasurface confirming the advantage of adding a birefringent substrate for the tunability of chiral metasurface. Figure S11(b) shows corresponding simulations, in very good agreement with the experiment.

**
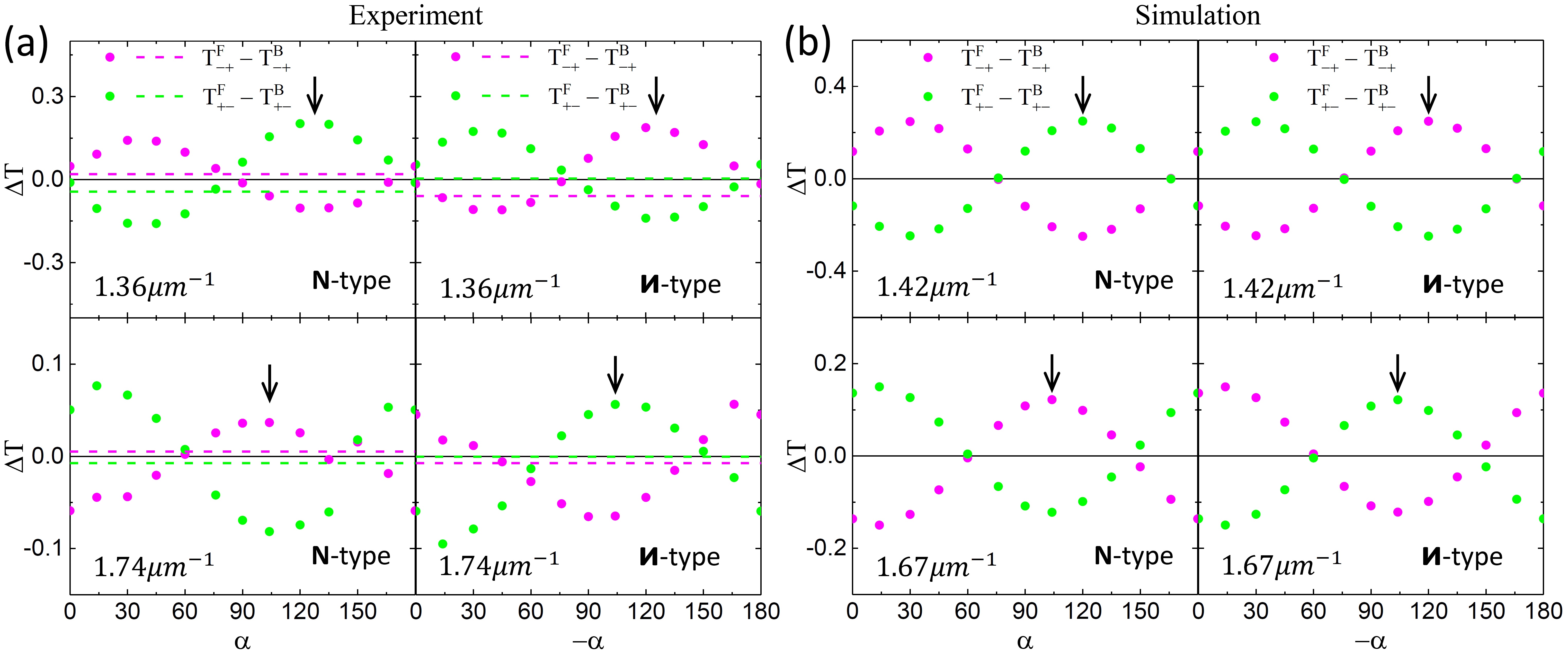
**

**Figure S11:** Asymmetric transmission of Au sawtooth metasurface.

(a) Cross-polarization asymmetric transmission for the **N**- and $\text{и}$-type Au sawtooth metasurface-sapphire system at various base angles at the resonances 1/*λ* ~1.36 μm^-1^ and 1/*λ* ~1.74 μm^-1^. The magenta and green dashed lines are values for the asymmetric transmission for the Au sawtooth metasurfaces without the sapphire slab. (b) Corresponding asymmetric transmission results from simulations for the resonances at wavenumbers 1/*λ* ~1.42 μm^-1^ and 1/*λ* ~1.67 μm^-1^. The black arrows in (a) and (b) indicate the maximum asymmetric transmission.

## References

[1] C. Menzel, C. Helgert, C. Rockstuhl, et al., “Asymmetric transmission of linearly polarized light at optical metamaterials,” Phys. Rev. Lett., vol. 104, p. 253902, 2010.

[2] R. R. Zhang, Q. L. Zhao, X. Wang, J. Li, and W. Y. Tam, “Circular phase-dichroism of chiral metasurface using birefringent interference,” Nano. Lett., vol. 20, pp. 2681-2687, 2020.

[3] R. R. Zhang, Q. L. Zhao, X. Wang, W. S. Gao, J. Li, and W. Y. Tam, “Measuring circular phase-dichroism of chiral metasurface,” Nanophotonics, vol. 8, pp. 909-920, 2019.

[4] G. X. Li, M. Kang, S. M. Chen, et al., “Spin-enabled plasmonic metasurfaces for manipulating orbital angular momentum of light,” Nano. Lett., vol. 13, pp. 4148-4151, 2013.

[5] Q. L. Zhao, T. Y. Yung, X. Wang, and W. Y. Tam, “Correction of numerical aperture effect on reflection phase measurement using thick-gap Fabry Perot etalon,” App. Opt., vol. 15, pp. 4392-4397, 2017.

[6] C. Menzel, C. Rockstuhl, and Falk Lederer, “Advanced Jones calculus for the classification of periodic metamaterials,” Phys. Rev. A, vol. 82, p. 053811, 2010.

[7] V. A. Fedotov, A. S. Schwanecke, and N. I. Zheludev, “Asymmetric transmission of light and enantiomerically sensitive plasmon resonance in planar chiral nanostructures,” Nano. Lett., vol. 7, pp. 1996-1999, 2007.

[8] T. K. Yung, Q. L. Zhao, W. S. Gao, X. Wang, and W. Y. Tam, “Measurement of reflection phase using thick-gap Fabry–Perot etalon,” App. Opt., vol. 55, pp. 7301-7306, 2016.

[9] W. S. Gao, C. Y. Ng, H. M. Leung, Y. H. Li, H. Chen, and W. Y. Tam, “Circular dichroism in single layered gold sawtooth gratings,” J. Opt. Soc. Am. B, vol. 29, pp. 3021-3026, 2012.
